# Amplification of the 20q Chromosomal Arm Occurs Early in Tumorigenic Transformation and May Initiate Cancer

**DOI:** 10.1371/journal.pone.0014632

**Published:** 2011-01-31

**Authors:** Yuval Tabach, Ira Kogan-Sakin, Yosef Buganim, Hilla Solomon, Naomi Goldfinger, Randi Hovland, Xi-Song Ke, Anne M. Oyan, Karl-H. Kalland, Varda Rotter, Eytan Domany

**Affiliations:** 1 Department of Physics of Complex Systems, Weizmann Institute of Science, Rehovot, Israel; 2 Department of Molecular Cell Biology, Weizmann Institute of Science, Rehovot, Israel; 3 Department of Medicine, Haukeland University Hospital, Bergen, Norway; 4 Department of Biomedicine, University of Bergen, Bergen, Norway; 5 The Gade Institute, University of Bergen, Bergen, Norway; 6 Department of Microbiology, Haukeland University Hospital, Bergen, Norway; Dana-Farber Cancer Institute, United States of America

## Abstract

Duplication of chromosomal arm 20q occurs in prostate, cervical, colon, gastric, bladder, melanoma, pancreas and breast cancer, suggesting that 20q amplification may play a causal role in tumorigenesis. According to an alternative view, chromosomal imbalance is mainly a common side effect of cancer progression. To test whether a specific genomic aberration might serve as a cancer initiating event, we established an in vitro system that models the evolutionary process of early stages of prostate tumor formation; normal prostate cells were immortalized by the over-expression of human telomerase catalytic subunit hTERT, and cultured for 650 days till several transformation hallmarks were observed. Gene expression patterns were measured and chromosomal aberrations were monitored by spectral karyotype analysis at different times. Several chromosomal aberrations, in particular duplication of chromosomal arm 20q, occurred early in the process and were fixed in the cell populations, while other aberrations became extinct shortly after their appearance. A wide range of bioinformatic tools, applied to our data and to data from several cancer databases, revealed that spontaneous 20q amplification can promote cancer initiation. Our computational model suggests that 20q amplification induced deregulation of several specific cancer-related pathways including the MAPK pathway, the p53 pathway and Polycomb group factors. In addition, activation of Myc, AML, B-Catenin and the ETS family transcription factors was identified as an important step in cancer development driven by 20q amplification. Finally we identified 13 "cancer initiating genes", located on 20q13, which were significantly over-expressed in many tumors, with expression levels correlated with tumor grade and outcome suggesting that these genes induce the malignant process upon 20q amplification.

## Introduction

Impaired genome stability is one of the hallmarks of cancer [1]. Local DNA copy number aberrations have been shown to be predictive of outcome [2], [3], [4] or of treatment response [5], [6], [7] in several cancers. Although most cancer cells exhibit gain or loss of chromosomal regions [8], there still is a debate between scientists whether genomic aberrations are essential for cancer initiation [9], [10] or an outcome of the tumorigenic process [11], [12], [13]. While some reports suggest that gain of an extra chromosome exerts anti-proliferative effects [14], [15], others claim that aneuploidy aberrations occur at a premalignant stage [10], [16], [17], [18] resulting in chromosomal variations and neoplastic phenotype [9].

Several chromosomal duplications have been frequently observed in many types of cancer. Among these, recurrent gain and amplification of the long arm of chromosome 20 (20q) has been observed in 90% of pancreatic cell-lines [19], 15–83% of pancreatic adenocarcinoma [20], around 70% of primary gastric cancers [21] and of colon cancer [18], [22], 50% of ovarian and cervical and 90% of breast [23] cancers. Gain of the 20q chromosomal arm was also shown to be a very frequent event at early stages of prostate carcinogenesis [24], [25]. In addition, gain of the 20q chromosomal region was noticed transiently in early passage stocks of human mammary fibroblasts, immortalized with hTERT and SV40 large-tumor oncoprotein [26]. Noticeably, in almost all these studies 20q is the most frequent amplification, and deletion of this arm is very rare. Furthermore, several studies show that amplification of 20q is correlated with poor prognosis [27], aggressive tumor phenotype, progression [28] and metastasis formation [19], [29], [30].

Assessing whether chromosomal imbalances play a causative role in tumorigenesis, as opposed to being bystanders is a difficult task. Studies done on clinical samples, of both chromosomal aberrations and gene expression, are hindered by a variety of confounding factors, which stem from different genetic backgrounds of patients, variable and uncharacterized mutations in tumors, and the uncontrolled contaminations by inflammatory, endothelial, and stromal cells. To overcome these obstacles, we previously established an in vitro transformation model based on the human lung fibroblasts, WI-38, which gave rise to the identification of gene expression signatures [31], [32], [33] associated with genetic aberrations [34]. In addition, human solid tumors are usually obtained from resections performed at a time when the tumor is already fully developed, which excludes the access to crucial information about tumor initiation and progression.

In order to obtain novel insights into the early stages of transformation and the genetic networks associated with chromosomal abnormalities, we used an in vitro model of prostate cellular transformation. In this model primary prostate epithelial cells which were previously immortalized by introducing the catalytic subunit of telomerase, hTERT (EP156T) [35] were grown in culture under controlled conditions. The specific aim of this study was to examine the hypothesis that a particular genomic aberration occurring at early stage of carcinogenesis is accompanied by changes in gene expression which could serve as a driving force for tumorigenesis. After 650 days in culture the EP156T derived cells shared several phenotypic, chromosomal and transcriptional attributes with prostate cancer samples. We report herein on two main findings. The first is the prominent and early role of 20q amplification in the development of our in-vitro cell culture towards a pre-malignant phenotype, with an enhanced proliferation rate. Second, we explain the malignant potential of 20q duplication by identifying 13 "cancer initiating genes”, located on 20q13, that are over-expressed in several kinds of cancer, and whose expression is correlated with tumor grade and outcome.

## Materials and Methods

### Preparation of cell lines

**Figure 1:**
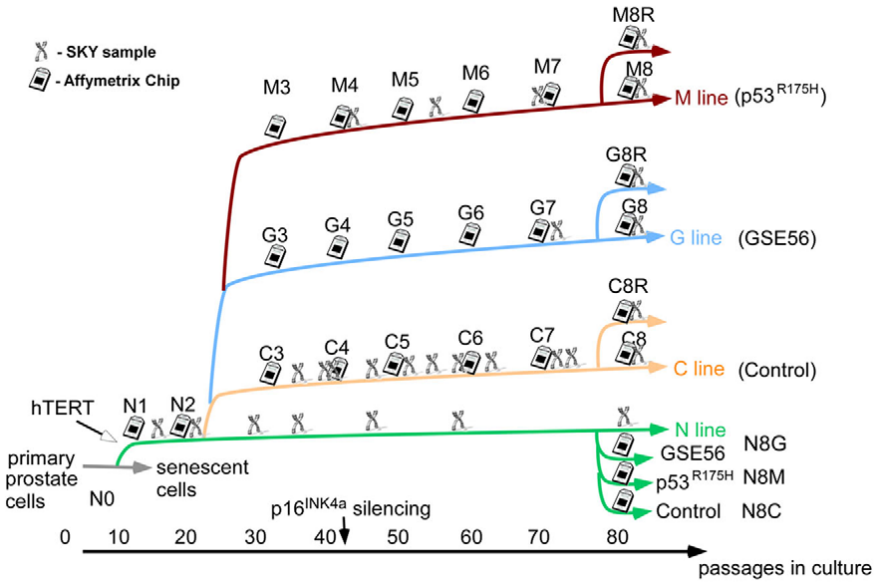
Establishment of the system consisting of four long term cultures. Schematic representation of the main derivative cultures of the EP156T prostate epithelial cells. Each line stands for the subculture generated in vitro by introduction of specific genetic modifications. The x axis represents the number of passages in culture (about one week per passage). The chip symbols represent points at which cells were collected and their RNA hybridized to microarrays; the code for the resulting sample, e.g. G5, represents the line (G) and the approximate number (50) of passages in culture divided by ten. The chromosome symbol indicates a SKY measurement.

Normal human prostate cells immortalized by telomerase introduction [35] were grown in culture for 650 days, during which they were analyzed for cell growth rate, chromosomal alterations and expression profiling. hTERT elongates chromosomal ends preventing replicative senescence of several kinds of normal human cells [36]. The hTERT immortalized EP156T cells were designated here as N line (Figure 1). The C, G and M lines were derived from the N line (Figure 1) after 25 passages (equivalent to about 150 days). To this end, a mutant of the tumor suppressor p53 (p53R175H) cloned into a retroviral vector PLXSN was introduced into the N line. The new cells, stably expressing the p53R175H mutant, were designated the M line. In parallel N cells were also infected with the PLXSN vector containing the GSE56 sequence encoding for a short peptide that inactivates the p53 tumor suppressor gene in a dominant negative manner [37], which gave rise to the G line. The empty PLXSN plasmid was used as a control generating the C line. Oncogenic H-RasV12 cloned into the pBabe-hygro retroviral plasmid was used for the infection of C, G and M lines just before passage 80 generating the C8R, the G8R and the M8R cells, respectively. Additional lines were created by the infection of late passage N cells with PLXSN plasmid encoding either the p53R175H mutant (N8M), the GSE56 (N8G) or with the empty plasmid (N8C) (Figure 1). The infection was followed by maintaining the cells for two weeks in the presence of 400 µg/ml Neomycin (for cells infected with the PLXSN vector) or with 50 µg/ml Hygromycin (for cells infected with the pBabe-hygro vector) in order to select for the stable expression of the introduced plasmids. The retroviral infection procedure is detailed in [32]. Growth conditions and media components are detailed in [35].

### Gene expression profiling along the evolution process of EP156T derived cultures

For gene expression profiling, samples were taken at 32 points along the cultivation process and hybridized with the GeneChip Human Genome U133A 2.0 Array (Figure 1). The microarrays were preprocessed using RMA [38] and normalized. All data is MIAME compliant; the raw data has been deposited in a GEO database. The GEO record number is- GSE23038. The 5000 most varying genes were sorted by the SPIN algorithm [39] and clustered using SPC [40] to identify 2 main stable clusters of genes with a correlated pattern of expression.

### Spectral Karyotype Analysis (SKY)

Exponentially growing cells were incubated with Colcemid (0.1 µg/ml) over night, trypsinized, lysed with hypotonic buffer, and fixed in glacial acetic acid/methanol (1∶3). The chromosomes were simultaneously hybridized with 24 combinatorially labeled chromosome painting probes and analyzed using the SD200 spectral bioimaging system (Applied Spectral Imaging Ltd., Migdal Haemek, Israel).

### S-phase analysis

Subconfluent cultures were labeled for one hour with 10 µM BrdUrd (Sigma). Cells were detached with trypsin, fixed in 70% ethanol, and treated as follows (PBS washes between each step): 2 M HCl and 0.5% Triton X-100 for 30 min at room temperature; 0.1 M Na_2_Br_4_O_7_ at pH 8.5; FITC-conjugated anti-BrdUrd (Becton Dickinson) diluted 1∶3 in PBS/1% BSA/0.5% Tween 20 for 1 hour at room temperature; and finally, 5 µg/ml propidium iodide and 0.1 mg/ml RNase A. Samples were analyzed by two-dimensional flow cytometry to detect both fluorescein and propidium iodide fluorescence using a fluorescence-activated cell sorter (FACS) (Becton Dickinson). At least 10,000 cells were analyzed per sample.

### Isolation of Total RNA

Total RNA for microarray experiment and for Quantitative Real Time PCR (QRT-PCR) was isolated using NucleoSpin RNA extract kit (Macherey-Nagel), according to the manufacturer's protocol.

### Quantitative Real Time PCR (QRT-PCR)

A 2 µg aliquot of the total RNA was reverse transcribed using MMLV RT (Promega) and random hexamer primers. QRT-PCR was performed using the SYBR-Green PCR Master Mix reagent (Applied Biosystems) on an ABI 7300 instrument (Applied Biosystems). The expression level for each gene was normalized to that of the GAPDH housekeeping gene in the same sample. The primers were designed using the Primer Express software. Primer sequences are available upon request.

### The Expression Karyotype

Expression data were used previously to infer chromosomal aberrations [41], [42]. Our working hypothesis is that changes in the copy number of the DNA of a full chromosome or part of it are reflected in the gene expression. Deletion of a chromosome causes, on the average, down regulation in the expression of the genes of that chromosome, while duplication should be reflected by a higher expression level. In order to identify chromosomal imbalances, we compared expression levels of the genes that reside on each chromosomal arm, in each sample, to a reference sample of normal cells (N0), and searched for significant differences in the expression levels. We introduced here a method for Chromosomal Imbalance Analysis, based on a paired t-test, to derive chromosomal copy number information from expression data. The Chromosomal Imbalance Analysis method is described, tested and compared with a previously derived technique [42] in the supplementary Text S1, see Figures S1 and S2.

### Comparison of the two computational methods with SKY

Two methods have been used to identify "expression karyotype" in samples. Using the Chromosomal Imbalance Analysis we calculated, for each sample *s*, a P-value for each chromosome to test if the mean difference between the genes in N0, compared to sample *s*, is different from zero. The binomial approach [42] determines whether a significant fraction of the genes on the chromosome is over-expressed in *s* versus N0 (or under-expressed). For details on the differences between the two approaches see supplementary Text S1.

The results obtained by SKY, which were used to estimate the predictive power of the two computational methods, were analyzed as follows. If a chromosomal aberration was observed in more than 50% of the karyotyped cells, it was considered as a true aberration; otherwise it was interpreted as noise and removed from the analysis. Using this definition of the SKY results as the "ground truth", we compared the performance of the two computational methods, using receiver operating characteristics (ROC) analysis (see Figure S2). The performances of the two methods were similar, and we adopted the Chromosomal Imbalance Analysis here.

### Chromosomal Imbalance Analysis correlation

Denote by *E_gs_* the expression level (after log and thresholding) of probeset *g* in sample *s*. For each probeset g in sample *s* we calculate Δ*E_gs_* = *E_gs_* – *E_g,_*_N0_. Calculate for each sample *s* the median of the Δ*E_gs_* for the significantly changing probeset from 20q (as defined in the supplementary Text S1 on Chromosomal Imbalance Analysis), to get *M_s_*. For each probeset *g* from 20q we calculate the Pearson correlation between the two sets of numbers: *E_gs_* and *M_s_*, and call this as the Chromosomal Imbalance Analysis correlation of probeset *g* (with the expression karyotype of 20q).

### Constructing the karyotype evolutionary tree

We constructed a "karyotype evolutionary tree" combining data from the SKY results and the expression karyotype. The normal karyotype "species" (46,XY) was the root/ancestor of all the evolved karyotypes. The tree was constructed manually using the data from both the expression karyotype and SKY. If the data from expression karyotype were not consistent with the SKY data at a specific point, we interpolated the behavior of two adjacent points, assuming that we were dealing with a continuous process.

### Copy number analysis

Ultra-high-resolution Affymetrix Genome-Wide human SNP arrays 6.0 were used to examine acquired genomic copy number changes and loss of heterozygosity of EP156T cells. Genomic DNA was purified using the Tissue DNAkit (Cat.#D3396-02, EZNA, OMEGA Biotek). DNA prehandling and array hybridization was performed according to the manufactures instructions (P/N 702504, Rev3, Affymetrix, Santa Clara, CA), and scanned in an Affymetrix GeneChip Scanner 3000. Quality control, genotype calling, probe level normalization and copy number normalization to produce log_2_ ratios were made in Affymetrix GeneChip® Genotyping Console v3.0.1. An in-house reference file generated from 59 healthy blood donors was used. Data analysis and visualization was performed in Chromosome analysis suite with a threshold of minimum 100 kb and 20 markers. Aberrations are reported according to ICSN nomenclature and NCBI build 36.

### Networks and Graphical Representations

Data were analyzed through the use of Ingenuity Pathways Analysis (Ingenuity® Systems, www.ingenuity.com). The network is a graphical representation of the molecular relationships between genes/gene products. Genes or gene products are represented as nodes, and the biological relationship between two nodes is represented as an edge (line). All edges are supported by at least one reference from the literature, from a textbook, or from canonical information stored in the Ingenuity Pathways Knowledge Base. Human, mouse, and rat orthologs of a gene are stored as separate objects in the Ingenuity Pathways Knowledge Base, but are represented as a single node in the network. Nodes are displayed using various shapes that represent the functional class of the gene product.

## Results

### An in vitro model for early prostate carcinogenesis

In order to follow the initial progression stages towards malignancy in prostate carcinogenesis, we have established a system based on hTERT-immortalized benign prostate epithelial (EP156T) cells [35]. These immortalized cells are designated here as N line. At passage 25, N cells were manipulated to over-express reagents that inactivate the tumor suppressor p53. To that end, p53R175H mutant of the tumor suppressor p53 cloned into a retroviral vector PLXSN was introduced into the N line in parallel with the GSE56, a p53-inactivating peptide, cloned into the PLXSN vector, and with an empty PLXSN plasmid as a control. The four immortalized cell lines including Normal (N), transduced with GSE56 (G), with mutant- p53R175H (M) and with an empty vector for control (C) were cultured for an additional 500 days in vitro. Figure 1 schematically represents this model. Throughout this manuscript, samples are denoted by LX, where L = N,G,M or C and the numeral X indicates the number of passages (±3) at which the specific sample was taken, divided by ten (e.g. G4 represent sample taken from the GSE line at passage ∼40). Just before passage 80, oncogenic Ras (H-RasV12) was introduced into the C, M and G lines yielding C8R, M8R and G8R lines, respectively as depicted in Figure 1. To this end, the H-RasV12 oncogene cloned into pBabe-Hygro retroviral vector was used. Additionally, the retroviral reagents described above were used to over-express the GSE56 and mutant p53R175H in the cells of the N line at a late passage, generating the N8G, N8M and N8C lines (Figure 1). A retroviral infection was followed by two weeks of drug selection to induce stable expression (see Materials and Methods). To obtain a comprehensive picture of the evolutionary changes during the prolonged cultivation of cell cultures, we wished to combine the analyses of cell growth rate, chromosomal alterations and expression profiling along 650 days in culture of the N, C, G and M lines. To attain this goal, cells were sampled along this period and the analyses of cell proliferation, gene expression and karyotype were performed (Figure 1).

**Figure 2:**
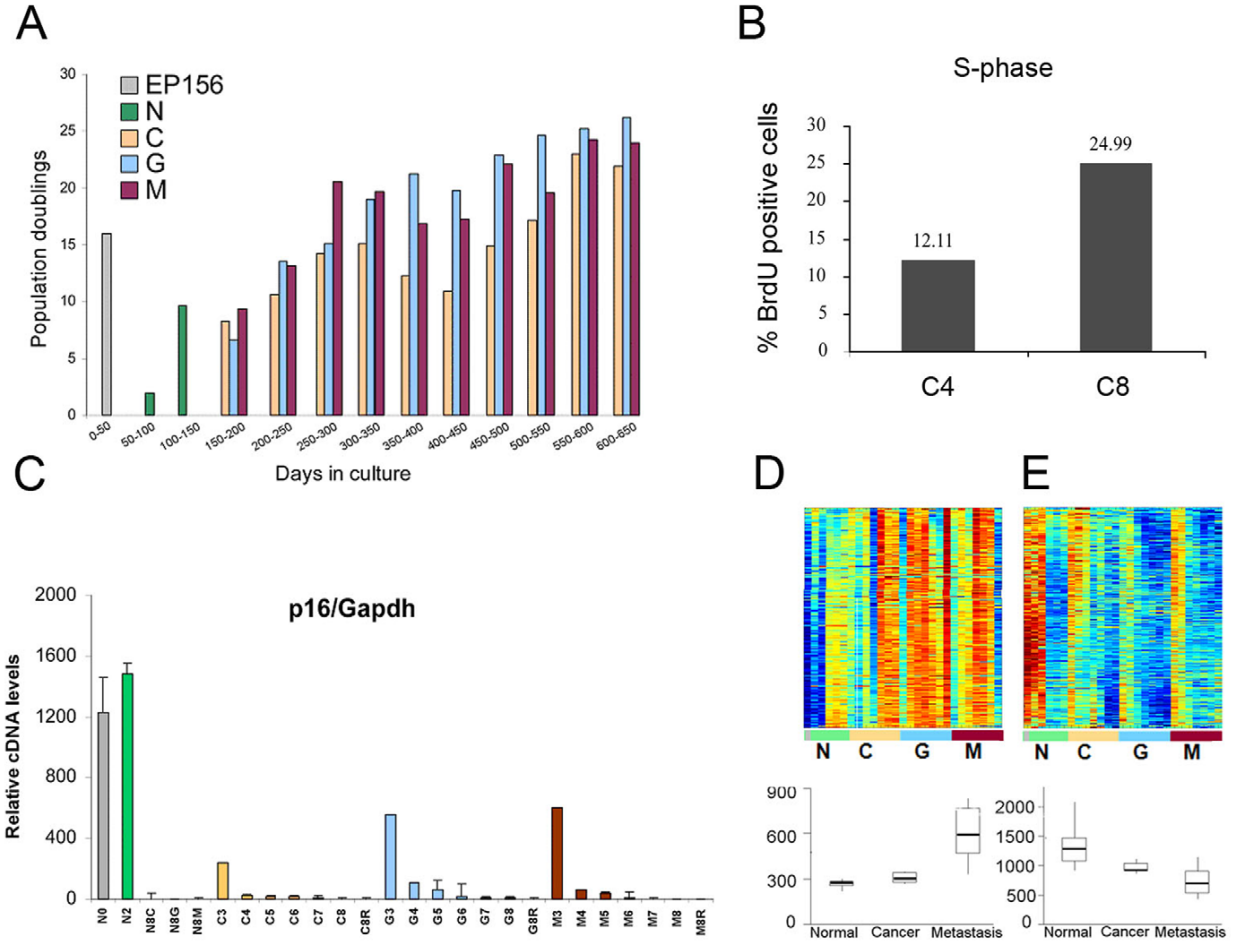
Late passages of EP156T sub cultures exhibit an increased growth pothential and a gene expression pattern resembeling that of prostate tumors. A. Number of population doublings of EP156T-derived cultures (C, G and M) calculated in 50-day intervals during the 650-day culture period after hTERT infection, alongside with the number of population doublings performed by non-infected EP156 cells in the first 50 days in culture and the EP156T (N) cells during the next 100 days in culture. B. Percentage of proliferating cells (S-phase) was measured by BrdU labeling of early passage (C4) and late passage (C8) cells. C. Real time QRT-PCR for p16INK4a expression in the different samples of EP156T system. D. Top: Expression matrix of a cluster of genes whose expression increased during the transformation process. Within each line samples are ordered from early to late passages. Lower panel: the mean expression pattern of these genes in samples from prostate cancer patients [45]. The p-value for the difference in expression between normal and cancer is p = 0.00024 and from normal and cancer to metastasis is p = 0.00013. E. Top: Cluster of genes containing over-represented glycoprotein and extracellular region genes, the samples for each line are ordered from early to late passages. The cluster is down-regulated during the transformation process. The lower graph presents the mean expression of the cluster's genes in cancer samples [45]. The p-value for the difference in expression between normal and cancer is p = 0.00013 and from normal and cancer to metastasis is p = 0.00023.

Upon the establishment of our in vitro transformation model, it was important to evaluate whether this system may represent in-vivo human cancer in a reliable manner. To that end, we examined whether along the 650 days of cultivation, the EP156T cells acquired molecular and phenotypic features typical of actual tumors. As cellular proliferation is the basic feature of cancerous transformation, cell growth rate and percentage of proliferating cells (i. e., cells at the S-phase of the cell cycle), were assessed. Indeed, an increase in cell growth rate was evident as the cells cultivation progressed (Figure 2A). In agreement with this, a higher proportion of proliferating cells was detected at later passages, as judged by BrdU labeling and FACS analysis (Figure 2B). Additionally, the expression of the p16INK4a tumor suppressor gene decreased with time in culture in all four cell lines (Figure 2C). Reduced levels of p16INK4a are commonly observed in advanced prostate tumors [43] and were found to be one of the central events in an in vitro transformation process of human WI-38 fibroblasts [31], [32], [33]. Then, we wished to examine whether the gene expression pattern of EP156T-derived cultures resembles that of in vivo tumors. To achieve this goal, the following analysis was conducted. Upon gene expression profiling of the EP156T cells, a clustering analysis was performed to identify the genes with correlated expression pattern. The expression patterns of the two most prominent clusters are presented in Figures 2D and 2E. The first cluster (Figure 2D) contained 177 transcripts that were up regulated over time in culture. When examining the Gene Ontology (GO) enrichment using the David software [44], we found that these genes were associated with cell proliferation. Thus, this cluster is termed the "proliferation cluster" along the manuscript. This trend of expression was validated by QRT-PCR on three representative genes (Figure S3). The second cluster (Figure 2E) contained 296 glycoprotein and cell adhesion molecule transcripts and their expression negatively correlated with the proliferation cluster. To evaluate whether these changes in gene expression can be related to the transformation process of EP156T cells, we examined the expression of the genes of both clusters in a dataset of 99 samples, obtained from normal prostate, tumor and metastasis of prostate cancer patients [45]. Strikingly, the expression of genes of both clusters significantly correlated with their expression in this set of clinical cancer progression samples (Figure 2D and 2E, bottom). The finding that, the gene expression pattern of our in vitro model resembles that of advanced prostate cancer, suggests that this model recapitulates the molecular events which occur during in vivo malignant transformation of the prostate gland. The detailed analysis of these clusters and the comparison to in vivo data is provided in Text S1. Together, these observations suggest that during prolonged in vitro culturing of EP156T prostate immortalized cells, a selection process took place favoring survival of cells with higher proliferative capacities which might lead to a transformed phenotype.

Exogenous introduction of telomerase induced the immortalization of EP156T cells due to telomere elongation which was evident after maintaining the cells for long period in vitro [35]. Furthermore, over-expression of the mutant form of the p53 tumor suppressor and of p53 inactivating peptide GSE56 were also maintained for long period after their introduction to the cells. A detailed analysis of the specific functions of mutant p53R175H over-expressed in EP156T cells is presented in [46]. The retention of telomerase and altered forms of p53 in EP156T cells over time suggests that these genes may play a role in the maintenance of transformation.

### 20q amplification emerged and dominated the cell population at early stages of the process

#### Chromosomal abnormalities

We turned to examine the chromosomal copy number variations in our cells, as these have been found to be related to cancer phenotypes. To this end, we estimated the chromosomal aberration pattern at the population level using computational analysis of expression data (see Methods), and on the level of single cells by means of spectral karyotyping (SKY). We used our data to follow the temporal evolution of the "expression karyotype"- i.e. the chromosomal aberrations, as they were reflected in the gene expression pattern. Our data constitute a real time series, which gives us significant advantages in identifying the progression of the dominant chromosomal aberrations.

**Figure 3:**
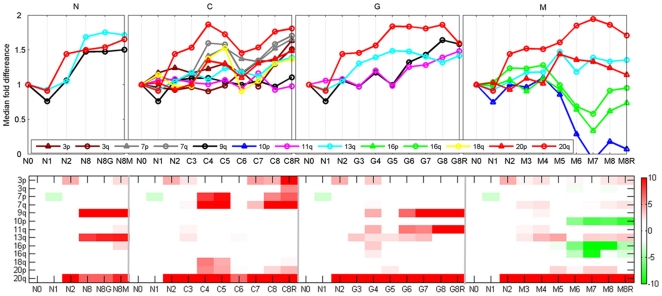
Copy number change from expression: results of the (Chromosomal Imbalance Analysis) algorithm. Expression level of each gene annotated to a particular chromosomal arm was compared to its expression level in the N0 sample (EP156 primary cells at passage 8) that represents the parental culture of all four lines. For each of the N, C, G and M lines (see text) the upper panel shows the coordinated changes in the median of the expression of genes annotated to specific chromosomal regions, divided by their median expression in N0. The lower panel is the –log _10_(p-value) of the paired t-test between the genes in N0 and the other samples (x-axis) on a specific chromosomal arm. The figure presents the statistically significant chromosomal arms (p-value<0.001 and median fold change >1.5 or <0.5 at least at one point).

The "expression karyotype" revealed significant temporal variation of the expression levels of the genes of several chromosomal arms for the different lines (Figure 3). Chromosomal arm 20q showed the most pronounced changes of expression and was associated with the most significant p-values in all the lines. When compared to N0, the expression of 20q showed about 1.5 fold increase in the N line and in the early passages of the M, G and C lines. Interestingly, in the late passages of the M, G, and C lines this fold change increased to ∼1.8. These results suggest that cells with a trisomy of 20q dominated the cell population at early stages of the process, and in the C, G and M lines secondary events followed and created more than 3 copies of the 20q arm. Additional aberrations that were observed were amplifications of 9q and 13q in the N line; of 7p, 7q, 18q and, later in the process, of 3p in the C line; amplification of 9p, 11p and, less significantly, of 13q in the G line; in the M line we identified, in addition to amplifications of 13q and 20p, also deletions of 10p and chromosome 16 (after passage 60).

We performed 24 SKY measurements at different stages of the process; in each we examined between 4–10 cells (the results are summarized in Table 1). SKY, in agreement with the expression karyotype, identified duplication of chromosomes 20, 3, 7, 9, 18 and deletion of 16 along the different lines. In addition, translocations involving chromosomes 10, 11 and 20 that were identified by SKY, were recognized as deletions/duplications by the expression karyotype (as will be discussed below).

**Table 1:** Karyotype analysis of EP156T derived cultures.

Name passage (chip)	# cells	karyotype
p18 (N2)	4	46,XY,dup(20)
p24 (N2)	4	46,XY dup(20)
p28	8	46,XY,dup(20)
p31	7	46,XY,dup(20)
p41	7	5/47,XY,+7,dup(20); 1/47,XY,+7,+9, dup(20); 1/48,XY,+7,+18,dup(20)
p52	7	6/47,XY,+dup(20); 1/46,XY,+dup(20),−21
p58	8	5/47,XY,+9,del(8),der(19)t(8;19),dup(20); 2/46,XY,+9, −21,dup(20); 1/47,XY,+dup(20)
p80 (N8)	6	45–47, XY,+9,der(8)t(8;13),dup(20) In several cells we found del(13) and del(5).
Neo p35 (C4)	8	49,XY,+7,+18,+dup(20)
Neo p39 (C4)	8	2/47,XY,+9,dup(20); 4/47,XY,+7,dup(20); 3/46,XY,+7,−21,dup(20); 2/46,XY,dup(20);
Neo p47 (C5)	8	5/47,XY,+7,dup(20); 1/48,XY,+7,+18,dup(20); 1/48,X0,+7,+18,dup(20); 1/47,XY,+7,+18,−22,dup(20)
Neo p53 (C5)	5	50,XY,+3,+7,+18,dup(20)
Neo p54 (C5)	6	1/50,XY,+3,+7,+18,dup(20); 1/48,XY,+3,+7,+18,+dup(20),−10,−21; 1/48,XY,+3,+dup(20)2/49,XY,+7,+18,dup(20),der(10)t(7;10); 1/49,XY,+3,+7,+dup(20),der(10)t(7;10);
Neo p62 (C6)	9	4/46,XY,dup(20); 5/45,XY,+3,+7,+18,+dup(20)
Neo p74 (C7)	6	5/50,XY, +3,+7,+18,dup(20); 1/49,XY,+3,+7,+18,+dup(20),−21
Neo p75 (C7)	7	6/50,XY,+3,+7,+18,dup(20); 1/46,XY, dup(20)
Neo p85 (C8)	9	6/48,XY,+7,+18,dup(20); 3/50,XY,+7,+18,dup(20)+3,+20.
Neo-Ras p82 (C8R)	10	50, XY,+3,+7,+18,dup(20)
GSE p87 (G8)	9	46,XY, der(9)t(9;20;9;11),der(20)t(9;20),dup(20)
GSE-Ras p88 (G8R)	7	46,XY, der(9)t(9;20;9;11),der(20)t(9;20),dup(20)
Mp53 p43 (M4)	7	4/46,XY,dup(20); 1/45,XY,−21,dup(20); 1/45,XY,−7, dup(20)1/46,XY,−18;der(4)t(4;18)+der(11)t(11;18),dup(20);
Mp53 p54 (M5-6)	8	3/45,XY,−16,der(10)t(10;20),dup(20); 1/44,XY,−22,−16,der(10)t(10;20),dup(20);1/43,XY,−22,−16,−12,der(10)t(10;20),dup(20); 1/45,X0,der(10)t(10;20),dup(20);1/44,XY,−16,−22,Del(3),der(10)t(10;20),der(14)t(3;14),dup(20);1/45XY,−14,−16,+7,der(7)t(7;14),der(10)t(10;20),dup(20);
Mp53 p67 (M6-7)	7	4/45,XY,−16,der(10)t(10;20),dup(20); 1/44,X0,−16,der(10)t(10;20),dup(20)1/43,X0,−16,−22,der(10)t(10;20),dup(20); 1/44,XY,−16,−22,der(10)t(10;20),dup(20)
Mp53 p82 (M8)	4	2/45,XY,−16,der(10)t(10;20),dup(20); 1/44,X0,−16,der(10)t(10;20),dup(20)1/84,XX00,−2X16,−2X18,−2X21,2Xder(10)t(10;20),dup(20)

First column contains the identifier of the passage at which the cells were harvested. If expression measurements were performed on the same samples, the symbol for the array is also indicated. Second column - the number of cells for which SKY analysis was performed. Third column: results of the SKY analysis. +/− indicate additional or missing chromosome. "der" denotes a derivative chromosome with translocation (t) from another chromosome (as specified in the parenthesis). "dup" - duplication of a segment in the chromosome. For example “−20” means deletion of chromosome 20, “20” – duplication of chromosome 20 and “dup20” means amplification of part of chromosome 20.

At several points the SKY results do not behave according to our expectations (Table 1). To perform SKY analysis, cells were defrosted and re-grown at several points rather than using exactly the same population that was taken for microarrays. Thus founder effects may be responsible for inconsistencies between different SKY results (e.g. the duplication of chromosome 7 in passage 41 is inconsistent with passages 31 and 52) and between SKY and the expression karyotype observed at some points (e.g. the duplication of chromosome 18 in the C line or the deletions of chromosomes 13 and 5 in sample N8).

#### The karyotype evolutionary tree

**Figure 4:**
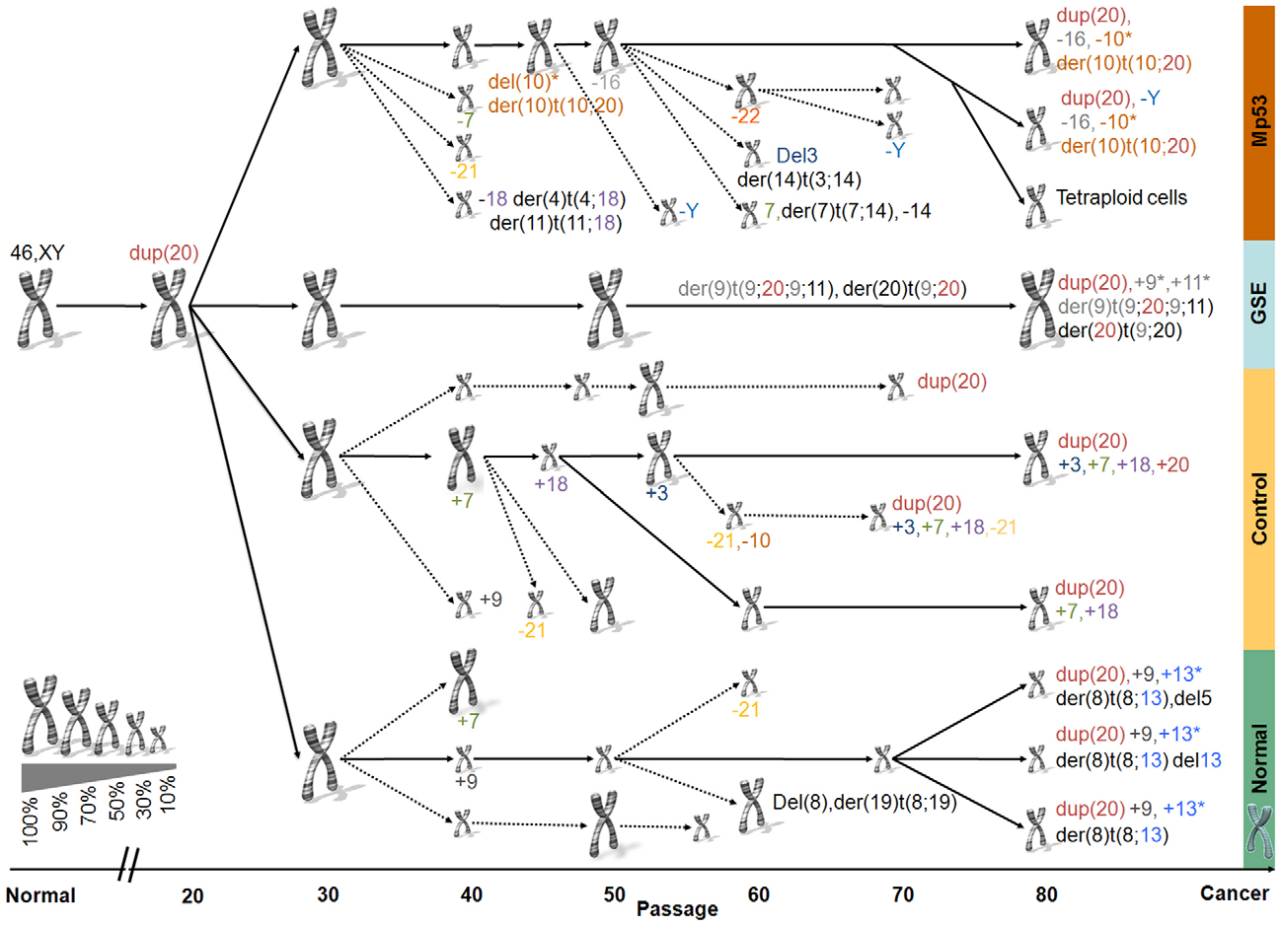
Karyotype evolutionary tree. The figure was generated on the basis of the 24 SKY results along the prostate cancer transformation process and the expression karyotype. Normal prostate human cells (left side of the figure) were used to establish 4 immortalized different lines: GSE, Control, Normal, and Mutant p53 (see text) that proliferate during 80 passages (x-axis). The chromosome sign denotes a karyotype, its size represents the percent from the total cells (larger size corresponds to higher percentage) and the colored number (if exists) under the chromosomal sign corresponds to a new aberration that appears. The dashed arrows represent karyotype "species" that become extinct. Since the SKY was done only on small numbers of cells and the cells were under selective pressure, in case of a discrepancy between different SKY results along the time line our assumption is that there is continuity in the cells. That is, an aberration has low probability to disappear and then appear again, rather than being missed in a single SKY. Similarly to other evolutionary trees, the "karyotype evolutionary tree" allows us to understand better which karyotype "species" emerge, evolve or become extinct during the process. The evolutionary tree points out the trait (in our case the specific insertion, deletion or aberration) that gives a particular karyotype growth advantage over others.

To understand better the evolutionary processes that took place in the course of our experiment, we constructed a "karyotype evolutionary tree" (Figure 4) combining data from the SKY results and expression. Similarly to other evolutionary trees, the "karyotype evolutionary tree" allows us to understand better which karyotype "species" emerge, evolve or become extinct during the process. The evolutionary tree points out the trait (in our case the specific insertion, deletion or aberration) that gives the selected karyotype growth advantage over other karyotypes. If the data from the expression karyotype were not consistent with the data from SKY analysis at a specific point, we interpolated the behavior of two adjacent points (see Methods). For example, the SKY results in the C line showed cells with combinations of additional amplifications and deletions. Although several possible constructions of the C line tree were possible, evidence from both the expression karyotype and from SKY suggested that the most dominant effect in these cells was duplication of chromosome 7 (at passage 40) followed by duplication of chromosome 18, which later was followed by duplication of chromosome 3. On the other hand the expression karyotype in samples C6 and C7 showed unexpected decrease in the median fold changes of all the aberrant chromosomes (see Figure 3). We believe that SKY represents more accurately the true karyotype at these time points.

#### The 20q amplification

Early in the process, after about 20 passages, cells that had extra material of 20q – denoted dup(20) – emerged, and from that point on dominated the cell population. In addition, three other translocations, all of which involved gain of additional chromosome 20 material: der(10)t(10;20) (denotes a derivate of chromosome 10 with additional material from chromosome 20), der(9)t(9;20;9;11) and der(20)t(20;9) were fixed in some of the populations and caused changes in gene expression (see Figure 3 and Figure 4). Contrary to this, we observed that deletion of chromosome 21 was detected several times along the in vitro transformation. Interestingly, in all these cases the karyotype species which contained deletion of 21 became extinct. This observation suggested that deletion of chromosome 21 was relatively frequent, but had adverse impact on evolutionary fitness. From the evolutionary tree it is evident that aberrations involving the 20q chromosomal arm are the most frequent to be fixed in the population, resulting in additional copies of genetic material from this region.

### The connection between karyotype and growth rate

The chromosomal imbalance analysis algorithms showed gradually and statistically significant changes (p-value<0.001 and median |fold| >0.5) in the "karyotype expression" along the transformation process (Figure 3). The most prominent results were observed for up regulation of genes encoded by chromosome 20q. Most strikingly, it is seen already in the N2 sample that corresponds to EP156T culture at passage 22. By SKY analysis, we validated that chromosome 20 aberrations appeared in N, C, G and M lines. This suggested that trisomy, partial trisomy or some other aberration that involved 20q, which occurred in the beginning of the transformation process, had an evolutionary advantage and as a result, this chromosomal aberration took over the entire cell population.

**Figure 5:**
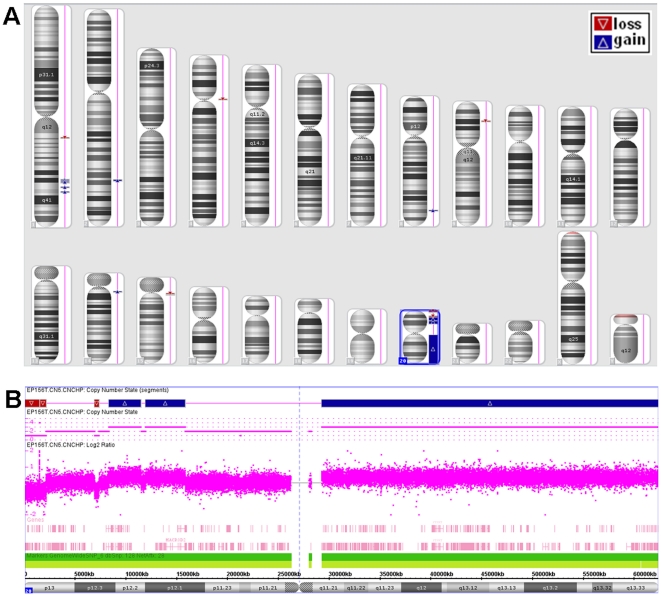
Chromosome copy number analysis (Affymetrix 6.0) of EP156T cells at passage 81. A. Regions of chromosomal gain (blue arrowheads) and loss (red arrowheads) are mapped onto the different chromosomes and chromosome 20 is highlighted. B. Magnified view of the regions of chromosomal gain or loss on chromosome 20.

To test the possible linking of chromosomal duplications/deletions, as measured in our "karyotype expression", to alterations in the cell proliferation rate, we examined the changes in cell growth rate and searched for their possible correlation with any chromosomal aberration. The proliferation rate of EP156T-derived cells was not steady and exhibited occasional increases and decreases along the growth progression axis (Figure 2A). Crisis and selection processes that commonly characterize immortalization can explain the apparent fluctuations in cell growth rate. The growth rate instability probably reflects clonal expansion in the mass culture where clones with different proliferation potentials exist concomitantly. Thus, we were interested to test whether acquisition of specific chromosomal aberrations contributed to cell growth. To this end, we performed correlation analysis between cell growth rate and gene expression on each chromosomal arm. The population doubling rate was calculated by counting the cells at the time point of the expression measurement and 3 days before. Our analysis indicated that the expression changes of the genes encoded by chromosomal regions 7q, 13q, 19p and 20q as derived from our Chromosomal Imbalance Analysis algorithm (see Methods), were significantly correlated (p<0.05) with the population-doubling rate (Figure S4). As expected, 20q showed the highest and most significant (p = 0.001) correlation. Finally, it was important to validate directly chromosome 20 copy number alteration. To achieve this, we performed chromosome copy number analysis of EP156T cells (N cells) at passage 81 utilizing the ultra-high-resolution Affymetrix Genome-Wide human SNP arrays 6.0 (Figure 5). This analysis, indeed, demonstrated that the 20q chromosomal region was amplified in the EP156T cells. Figure 5B demonstrates that these cells contained one extra copy of the genomic material located between 20q11.22 and 20q13.33, a region that constitutes almost the entire 20q chromosomal arm. This direct analysis confirming genomic 20q amplification validates and supports our conclusions based on SKY and computational Chromosomal Imbalance Analysis.

#### Evidence from experiments on another cell type

We showed previously in a fibroblast in-vitro transformation system, that hTERT-induced immortalization of WI-38 human diploid fibroblasts results in the spontaneous emergence of rapidly proliferating variants (WI-38/Tfast) [32]. Although our previous karyotype analysis identified only a small translocation between chromosomes X and 17 [32], we decided to revisit this analysis using array CGH to search for additional chromosomal aberrations that might have been undetected previously. We tested cells from 3 time points along the 650-day-long WI-38 transformation process. For the array CGH we used cells harvested at a short time after immortalization (passage 34), cells from passage 70 that on average proliferate at a rate of 0.6 PDLs/per day, and highly proliferating cells at the end of the process (passage 93) that proliferate at 1.1 PDLs/per day on average. Importantly a careful look reveals a single chromosomal change that happened at the transition point from slow to fast growing cells. Increased expression (of about 25%) of the 20q genes was observed at passage 93 (Figure S5). These data, together with the high frequency of 20q amplification found in several cell lines [47], [48] and cancers support our assumption that amplification of 20q is a key factor in increasing cell proliferation rate.

### A model for cancerous transformation induced by 20q amplification

The observations made above suggest that the 20q amplification might have an important role in promoting cancer. In order to substantiate this claim, we developed a new methodology that combines data from four sources: our experiment, the Oncomine database [49], expression data from a study of prostate cancer patients with full clinical annotation [45] and data from six different array CGH cancer datasets. We present here a brief overview and outline (see Figure S6) of our methodology, focusing on the results of immediate biological relevance; a detailed explanation of our method can be found in the supplementary Text S1.

#### Identifying 13 genes on 20q13 that have high malignant potential

**Figure 6:**
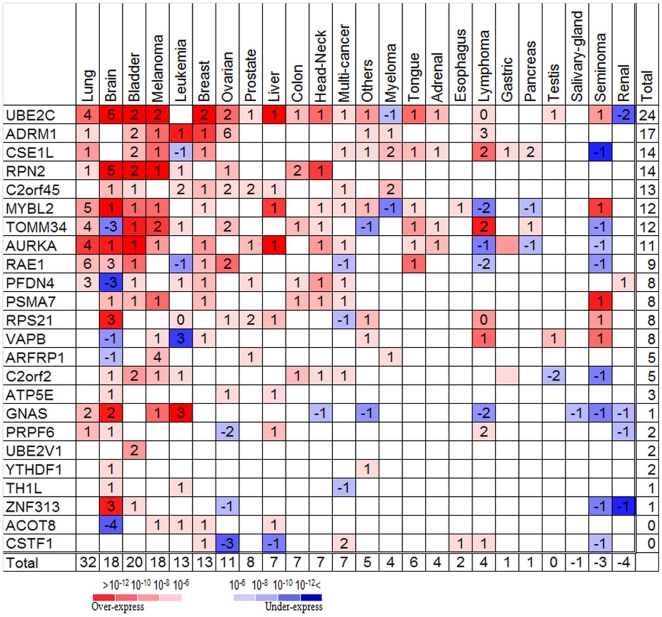
Summary of the expression of our 24 "purified primary target genes" in more than 360 experiments of different cancers. The colors represent the p-value (as calculated by Oncomine) for the significantly over-expressed (red colors) or under–expressed (blue colors) genes in cancer samples compared to normals. If there are *k* experiments (represented by the number *k* in the colored boxes) the colors represent the best of the *k* p-values. In the few cases when the gene was over-expressed in some experiments and under-expressed in others in the same kind of cancer, the number represents the differences between the two; positive numbers mean more experiments with over-expression, negative numbers - more under-expression.

Our working hypothesis is that one or several genes from 20q could serve as "cancer initiating genes" and impose tumorigenic effects on cells when over-expressed as a result of chromosomal amplification. To determine which genes are most likely to have this effect we applied several filters on the genes found on 20q. First we tested which of the genes located on 20q exhibited altered expression following the chromosomal aberration. We found 132 probesets on 20q, such that their expression was correlated, probably as a direct outcome, to 20q amplification (see Methods on Chromosomal Imbalance Analysis correlation and supplementary Text S1). We referred to these probesets as "primary target genes". Next, using six independent aCGH data sets [19], [21], [23], [50], [51], [52] we identified 20q13 as the region that was most commonly amplified in cancer. In addition, using expression data from 99 in vivo normal and cancerous prostate samples [45] (see figure S7 and supplementary Text S1) we narrowed down the number of probesets from 132 to 34. These correspond to 24 "purified primary target genes" located on 20q13, correlated with 20q amplification and were found to be relevant in cancerous prostate samples. We analyzed (see supplementary Text S1) the expression profile of each of the 24 "purified primary target genes" (Figure 6) using Oncomine [49], a compendium of expression data from 25,812 microarrays measured for 361 cancer data sets. Thirteen out of the 24: UBE2C, ADRM1, CSE1L, RPN2, C20orf45, MYBL2, TOMM34, AURKA, RAE1, PFDN4, PSMA7, RPS21 and VAPB showed exceptionally significant over-expression in several cancers, in progressed malignant stages (versus early) and in poor (compared to good) prognosis samples (see Figure S8 for stage, grade and prognosis and Figure S9 for survival data). We propose that these 13 "cancer initiating genes" are key players in the cancer-driving processes associated with 20q amplification.

#### Several Cancer-related pathways are controlled by 20q Amplification

If our hypothesis that 20q can promote cancer progression is true, a valid assumption would be that the "cancer initiating genes" can induce cancer by regulating, directly or indirectly, several cancer-related pathways. Therefore, we tried to find hints for the pathways that might be involved in these processes. As a first step, we tried to identify genes that might be regulated by genes on 20q. We identified 407 probesets of "secondary target genes" whose expression levels correlated with 20q amplification but located off the 20q arm. As such, their expression is also likely to be influenced indirectly by the amplification of 20q, probably by the "cancer initiating genes". These 407 probesets can indicate which processes happened in the cells as a result of the 20q amplification.

So far we did not try to answer either of the following questions: 1. How does over-expression of the "cancer initiating genes" cause up-regulation of the "secondary target genes"? 2. How does increased expression of the "secondary target genes" induce cancer progression? In order to bridge this gap, we performed a very wide scale search, using the Oncomine database, to identify which pathways might have been influenced as a result of the 20q amplification. We found that genes that changed as a result of MYC amplification in cancer had a high overlap with our "secondary target genes" (p-value 10^−59^). In addition, we found significant overlaps with genes over-expressed as a result of activities of Mutant *p53*, *MLL* and *ALL,* and with the loss of activity of Estrogen Receptor, *TEL-AML* translocation, B-Catenin, *ERBB2* and other "potential regulator genes" that are found in Table S1, and summarized in Table 2.

**Table 2:** Cancer related genes associated with 20q amplification.

Genes/Aberrations	# over -express	# under-express	p-val over	p-val under	Network
ABL-BCR	1	0	8.9E−06	---	RTK
B-Catenin	0	4	---	6.00E-20	APC
Bcl2	0	1	---	2.7E−18	APOP,p53, FLT3 signaling
BCL6 mutation	0	1	---	4.7E−06	
Bmi-1	0	1	---	3.9E-10	Polycomb
BRAF mutant	1	0	9.6E−07	---	RTK
BRCA1 mutant	1	0	1.3E−15	---	p53
CEBPA mutant	1	0	4.3E−21	---	
c-Src	2	0	5.6E−21	---	cytoskeleton
E2F3	0	1	---	5.6E−21	RTK, p53
EED	2	0	---	2.0E−06	Polycomb
EGF	1	2	4.3E−11	2.7E−18	RTK, EGF pathway
EGFR	1	2	0.0000086	2.7E−18	RTK, EGF pathway
ERBB2 (HER2)	1	4	0.0002	2E−11	RTK
Estrogen Receptor	1	10	1.1E−05	2.7E−19	
EVI1	0	1	---	4.2E−11	fussion with ETV6 (ETS)
EZH2	2	0	---	3.5E−14	Polycomb
FLI1	6	0	7.6E−11	---	ETS
FLT3	1	0	5.3E−10		FLT3 signaling
FLT3 Mutation	0	1		3.6E−08	
HIF-1	0	1	---	5.1E−13	HIF1
IFN-alpha	0	1	---	6.2E−09	Cytokine Network
IL-10	0	3	---	1.4E−15	Cytokine Network
INK4a Deletion	1	0	5.5E−05	---	p53
MEK	0	2	---	7.5E−23	RTK, Acitvate ETS
MLL	4	0	4.8E−35	---	EGF pathway, FLT3 signaling
Myc	8	0	3.8E−59	---	EGF pathway
NF1	1	0	1.9E−07	---	RTK
Notch blocking	0	7	---	9.8E−13	p53
p53	3	2	3.1E−13	9.8E−12	p53
p53 mutant	4	1	3.6E−15	4.3E−06	p53
PAX3-FKHR	0	1	---	3.5E−06	
PIK3CA mutant	0	1	---	8.3E−07	PI3K
PLZF/RAR	0	1	---	4.3E−35	
PML/RAR	0	1	---	0.0000016	
PTEN	0	2	---	0.000018	PI3K
Raf	0	2	---	0.0000043	RTK FLT3 signaling
RELA	0	1	0.000034	---	EGF pathway
SDHB	1	0	3.6E−08	---	HIF1
SUZ12	2	0	---	0.000036	Polycomb
SV40	2	0	7.5E−20	---	
TCF3/PBX1	1	0	0.0000023	---	
TEL-AML	0	5	---	1.1E−31	
VHL	0	1	---	2.6E−12	HIF1
Vitamin D	0	1	---	2.9E−33	

First column – list of "potential regulators". Second/third columns: the number of concepts that were over-expressed/under-expressed in the comparisons that defined the corresponding Oncomine concept. Each listed concept is associated with a "potential regulator" gene, and has a statistically significant overlap with the list of "secondary target genes". Columns four/five present the corresponding p-values (if more than one concept was based on a "potential regulator", the best p-value was cited). The last column shows the network to which the "potential regulator" belongs, as identified by [13].

**Figure 7:**
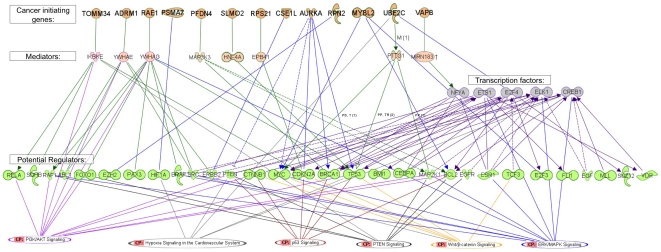
Cancer transformation model. Using Ingenuity Pathway Analysis (IPA, see Methods), we summarized the known regulatory interaction of "cancer initiating genes" with the "potential regulator genes" and transcription factors (blue arrows). When no direct connection was found, we searched for indirect connections through mediator genes. If more than one such gene was found, we connected representative interactions (green arrow). Finally, we connected the genes in the lists to known cancer related pathways, based on the IPA database. First row: the list of "cancer initiating genes" which are located on 20q13. Second row: mediators - genes that have protein-protein interaction with the "cancer initiating genes" or are regulated by these genes and are also known regulators of the "potential regulator genes". Third row: The transcription factors that were found by POC. 4th row: the "potential regulator genes". 5th row: the list of cancer pathways as defined by IPA and the "potential regulator genes" from our analysis that belong to these pathways. We filtered out the"potential regulator genes" and transcription factors for which no direct or through one mediator connection to our "cancer initiating genes" were found.

To understand better how the "potential regulators" might promote cancer, we characterized the pathways and networks which they regulate and to which they belong. GO analysis, using David software [44], of our "potential regulator genes" found enrichment of positive or negative regulation of cellular process (p- value = 2.6E−18 and 1.2E−14), cell proliferation (p = 2E−16), cell differentiation (2e−13), cellular developmental process (2e−13), regulation of apoptosis (2e−13). KEGG analysis [53] of the "potential regulator genes" found the prostate cancer pathway as the most significant pathway (1.1E-13) in addition to other cancers like bladder, pancreatic cancer, lung, leukemia and more (p = 3.6E−13 to 2.8E−5). Other significantly enriched KEGG pathways included ERBB (p = 2.6E−9), focal adhesion (p = 4.0E−7) and MAPK signaling (p = 1.5E−3). Finally we used Ingenuity Pathway Analysis (IPA) (see Methods) to assign each "potential regulator gene" to a biological pathway (Figure 7).

In order to produce a model for cancerogenesis induced by 20q amplification, we searched for known interactions between our "cancer initiating genes" and "potential regulator genes". We tried to identify transcription factors that were involved in the process and, finally, to connect all the components ("cancer initiating genes", "potential regulator genes" and transcription factors) to cancer related pathways. Promoter analysis of the "secondary target genes" using the POC software [54] identified a list of transcription factors. The most significant motifs that were found in our analysis were the ETS family transcription factors (like ELK1, FLI1 and others) and HIF1. Note that both HIF1 and ETS family transcription factors, also belong to our "potential regulator genes". In addition we found several significant (FDR 0.01) cell cycle related transcription factors like E2F, NFY and CDE, and few more motifs (see Table S2) in the promoters of the "secondary target genes". The lists of "cancer initiating genes", "potential regulator genes" and transcription factors were processed using the Ingenuity Pathway Analysis (IPA), scanning for known biological connections between the members of the lists (see Methods). Figure 7 summarizes the known regulatory interactions of "cancer initiating genes" with the "potential regulator genes" and transcription factors (blue arrows). If no direct connections were found, we searched for indirect connection through mediator genes, and if several such genes where found, we connected representative interactions (green arrows). Finally, we connected the genes in the lists to known pathways, based on the IPA database.

## Discussion

This study presents a detailed description and analysis of the changes in genome-wide expression and chromosomal karyotype, followed in the course of a unique 650-day long in vitro process, which transformed normal prostate cells into pre-cancer-like phenotypes. Chromosomal aberrations were identified in two independent complementary ways: directly, by SKY and indirectly, from gene expression data. Furthermore, we confirmed 20q amplification, the central chromosomal aberration in this system, by high resolution DNA copy number analysis using SNP arrays. We introduced and applied a novel approach to determine which biologically relevant networks were involved in the cancerous transformation process. To do this, we used extensive data from more then 300 previously published experiments from several data bases and a wide variety of bioinformatic tools.

Based on our results, we propose the following model for cancer initiation driven by 20q amplification (Figure 7): 1. Spontaneous amplification of 20q occurred in a subclone of cells and caused up-regulation of large group of genes located on this chromosomal arm. We identified 13 out of the 600 genes on chromosome 20 as those with high oncogenic potential. 2. The high expression levels of these genes caused direct and indirect changes in the activities of various transcription factors and oncogenic pathways. 3. The activation of those pathways and transcription factors caused increased activity of the cell cycle, metabolic and ribosomal pathways, and down-regulation of cell adhesion associated genes. These modifications of the cells' expression profile may have caused higher proliferation rate and loss of contact inhibition in early stages, progression of cancer, bad prognosis and aggressiveness in later stages. Indeed when we and others examine the expression pattern of 20q genes, and, in particular, of our 13 "cancer initiating genes" in different tumors, we find significant correlation with progression, tumor grade and outcome.

Our in-vitro model provided us a unique opportunity to monitor continuously the spontaneous evolutionary changes that happened during the 650 days of culture following immortalization. We established the amplification of chromosomal arm 20q as a key initiating event in early cancer formation. We identified 13 genes, located at the 20q13 chromosomal region, which are likely key players, driving progression in many cancers. Furthermore, our study suggests possible mechanisms through which amplification of an entire chromosomal region induces the cancerous transformation, providing clues that might explain why 20q amplification is one of the most common genetic aberrations in cancer.

Some previously identified potential oncogenes [55], [56] that also reside on 20q13 were not included: EEF1A2, PTPN1, and ZNF217 did not pass our filters. A possible reason for this might be that our primary filters were based on prostate cells; and unequal representation of different cancer studies in the Oncomine database that we used.

It will be important to further investigate and validate the crosstalk between the 13 genes on 20q13 and each of the suggested networks. It will be interesting to apply our method to other common chromosomal aberrations like 18q or 13q amplifications, but it is not clear whether a similar system can be found in which one of these amplifications is the central early alteration.

Different types of human cells might require either similar or different clonal markers of transformation. Interestingly, human fibroblasts immortalized by hTERT and the oncogenic sequences of the SV40 virus exhibited a transient gain of the genetic material from the chromosomal arm 20q [26] while the hTERT-immortalized EP156T cells demonstrated stable 20q amplification. This might suggest that the amplification of the 20q chromosomal region in the fibroblast cells containing SV40 large tumor oncoprotein was important for the transition point at which normal cells become immortal serving as an initiating event. The difference between these cells and our immortalized EP156T cells is that the immortalized fibroblasts contain SV40 large tumor oncoprotein and represent fibroblastic cells while our immortalized culture does not contain SV40 sequences and represents cells from epithelial origin. For these reasons EP156T cells may depend on the 20q amplification for their immortalized status.

In conclusion, we showed that 20q amplification occurred early in the transformation process and had a strong evolutionary advantage in the cell population. We proposed a model that explains the main routes through which 20q amplification may induce tumor initiation. Additionally, we suggested several potential oncogenes that might be key factors in this process. We believe that our work is an important step in understanding cancer initiation and progression and may give rise to new therapeutic possibilities by targeting the cause or the consequences of specific chromosomal aberrations.

## Supporting Information

Figure S1 Using our gene expression data we applied paired t-test to predict chromosomal aberration in all chromosomal arms in the 27 samples. The analysis was done using varying number of genes, from all (12000 unique genes) to the 1000 most variable genes. The results showed that using the 7500 to 5500 most variable genes (black box) maximizes for the number of predicted chromosomal duplications/deletions. This result does not dependent on the p-value that we chose as threshold.
(8.76 MB TIF)

Figure S2 ROC curves for the two methods. The x-axis represents the false-positives rate, and the y-axis represents the true-positives rate of the two methods, when compared to the SKY results.
(4.40 MB TIF)

Figure S3 QRT-PCR validation in proliferation cluster genes: BUB1 TPX2 and MYBL2 at progressive time points along the long term in vitro culture.
(3.01 MB TIF)

Figure S4 Pearson correlation between the growth rate and the expression of genes on each indicated chromosomal arm. * p-value<0.05, ** p-value<0.001 for up regulation of genes encoded by this region.
(0.69 MB TIF)

Figure S5 Raw array CGH data for WI-38 human diploid fibroblast cells obtained from passage 93 in an in-vitro transformation experiment (Milyavsky et al. 2005). Around 25% percent of the cells contained amplification of chromosome 20.
(0.24 MB TIF)

Figure S6 Workflow of our analysis of the tumorigenic effect of 20q amplification. The colors represent four different data types that were used: Oncomine (green), our prostate in-vitro transformation model (red), prostate samples from patients (blue) and array CGH data (black). Each box represents a step in the analysis. The arrows indicate that information was passed from one box to another.
(0.09 MB TIF)

Figure S7 Expression pattern of 20q genes in normal and cancerous prostate samples. Median Expression pattern (after normalization and log2) of genes in 99 in-vivo normal and cancerous prostate samples (Glinsky et al. 2004) at different stages of progression, as indicated by the color bar (blue for normal prostate, orange for primary tumor, red for metastatic tissue). A. Median expression of our "primary target genes". The blue dots mark samples which we predict to have 20q amplification B. Median expression of the "secondary target genes". C. Median expression of the genes encoded by the 20p chromosomal region. D. Median expression of the 20q genes which were not identified as "primary target genes" (not correlated with our 20q expression karyotype).
(2.91 MB TIF)

Figure S8 Summary of the expression of our 24 "purified primary target genes" in more than 360 experiments of different cancers. The colors represent the p-value (as calculated by Oncomine) for significant over-expression (red colors) or under-expression (blue colors) in different grade, stage or prognosis of each single "purified primary target genes". If there are several experiments (denoted by the number in the corresponding box) the color represents the best p-value from these experiments.
(8.32 MB TIF)

Figure S9 Survival and relapse test for our "cancer initiating genes" in several data sets. The figure shows genes whose expression levels (high 50% vs. low) significantly differentiate cancer patients by survival and relapse in several studies
(4.81 MB TIF)

Table S1 Oncomine Concepts with significant overlap with the "secondary target genes"
(0.14 MB XLS)

Table S2 List of over represented motifs (that pass FDR of 0.01) in the "secondary target genes" promoters. We ran promoter analysis on the two "secondary target genes" groups (that passed the filters R>0.7 and R>0.6, see text). (R>0.6) denotes that the motif was found only when we used correlation R>0.6. Conserved in mouse - The motif is conserved in the mouse promoters
(0.04 MB XLS)

Text S1 Supplementary information
(0.16 MB DOC)
