# Supplementary figures and images for: Amplification of the 20q Chromosomal Arm Occurs Early in Tumorigenic Transformation and May Initiate Cancer
Source: PLoS One. 2011 Jan 31;6(1):e14632. doi: 10.1371/journal.pone.0014632 (PMC3031497; doi:10.1371/journal.pone.0014632)

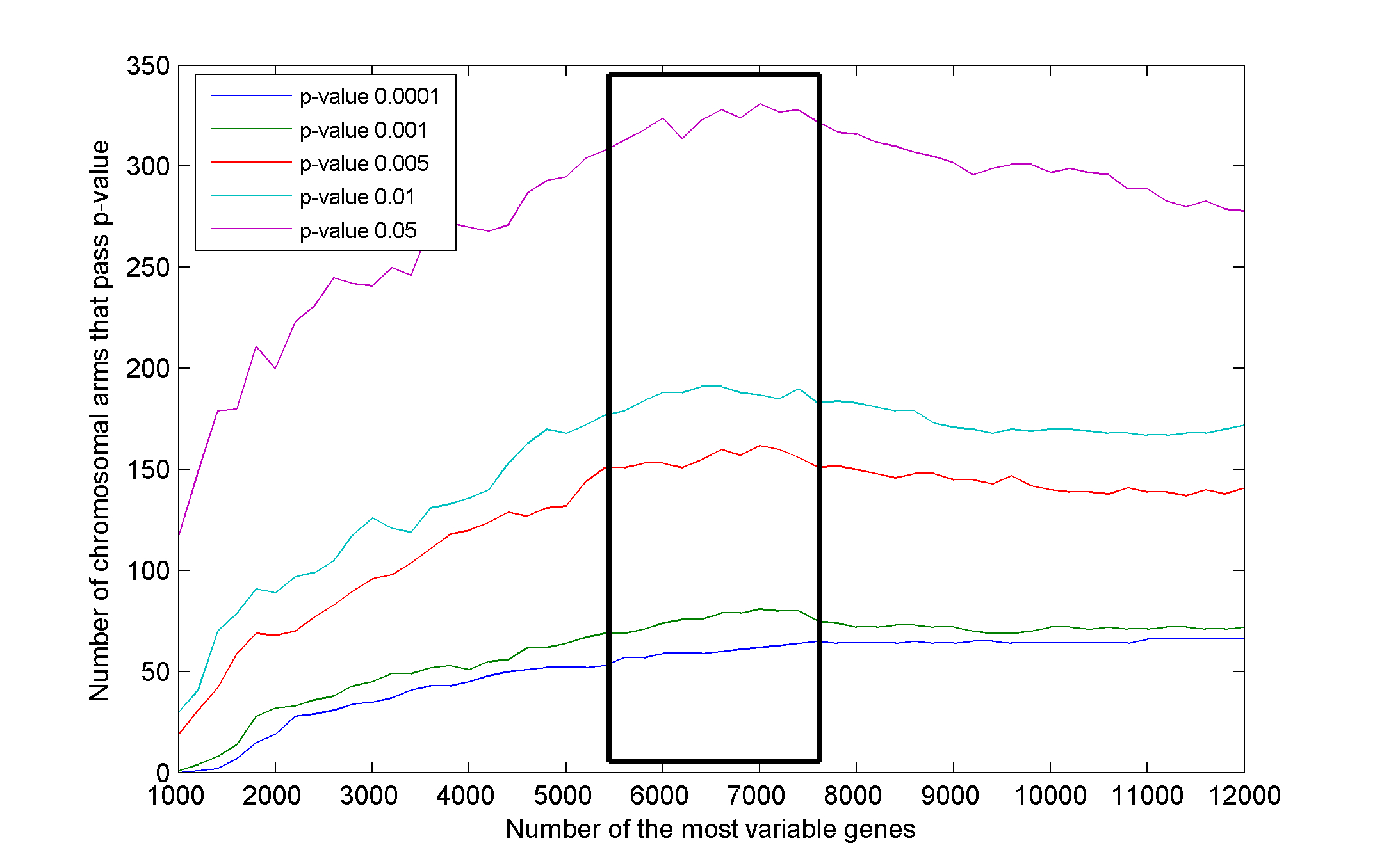

Supplement: Figure S1 — Using our gene expression data we applied paired t-test to predict chromosomal aberration in all chromosomal arms in the 27 samples. The analysis was done using varying number of genes, from all (12000 unique genes) to the 1000 most variable genes. The results showed that using the 7500 to 5500 most variable genes (black box) maximizes for the number of predicted chromosomal duplications/deletions. This result does not dependent on the p-value that we chose as threshold. (8.76 MB TIF) [file pone.0014632.s001.tif]

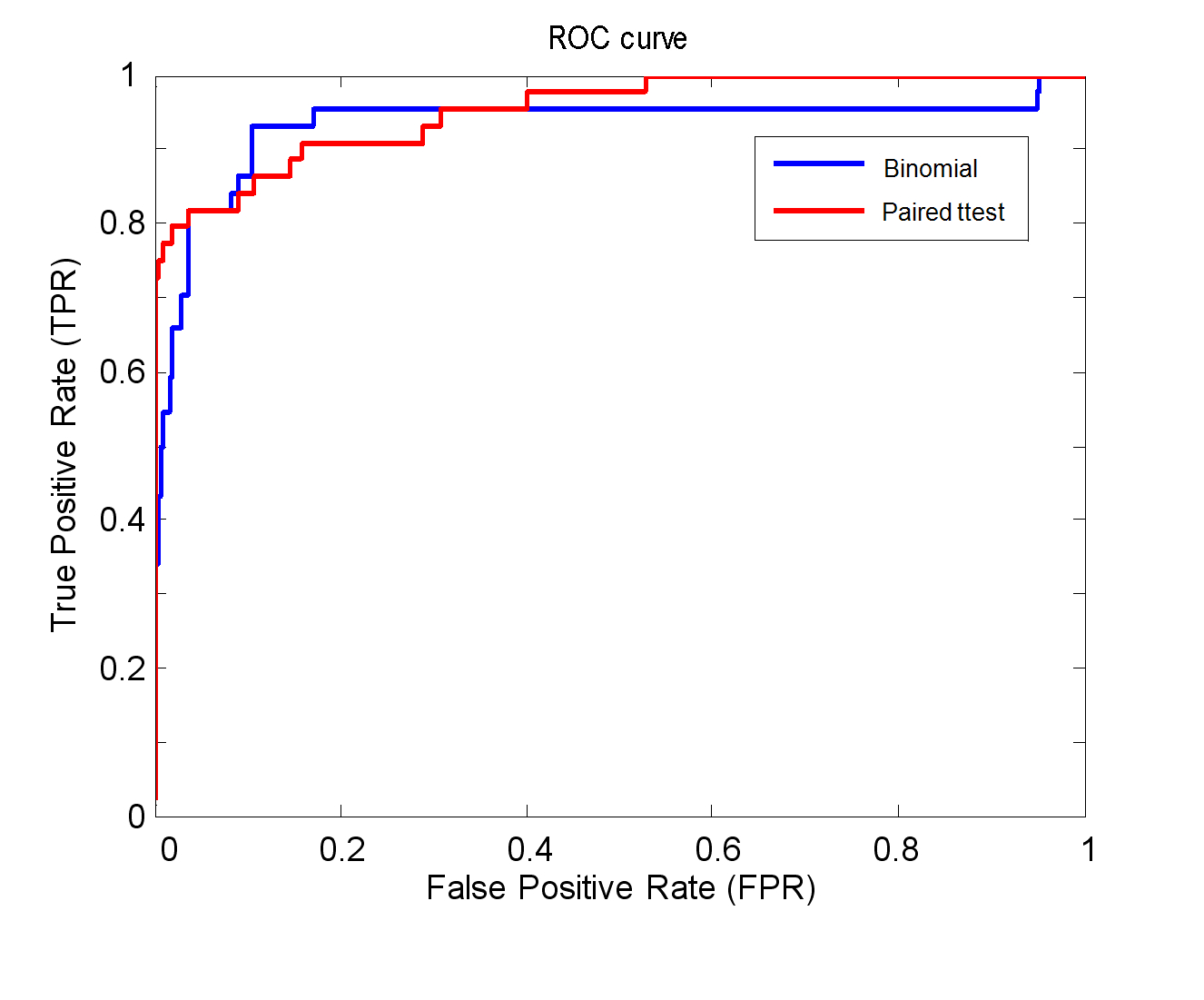

Supplement: Figure S2 — ROC curves for the two methods. The x-axis represents the false-positives rate, and the y-axis represents the true-positives rate of the two methods, when compared to the SKY results. (4.40 MB TIF) [file pone.0014632.s002.tif]

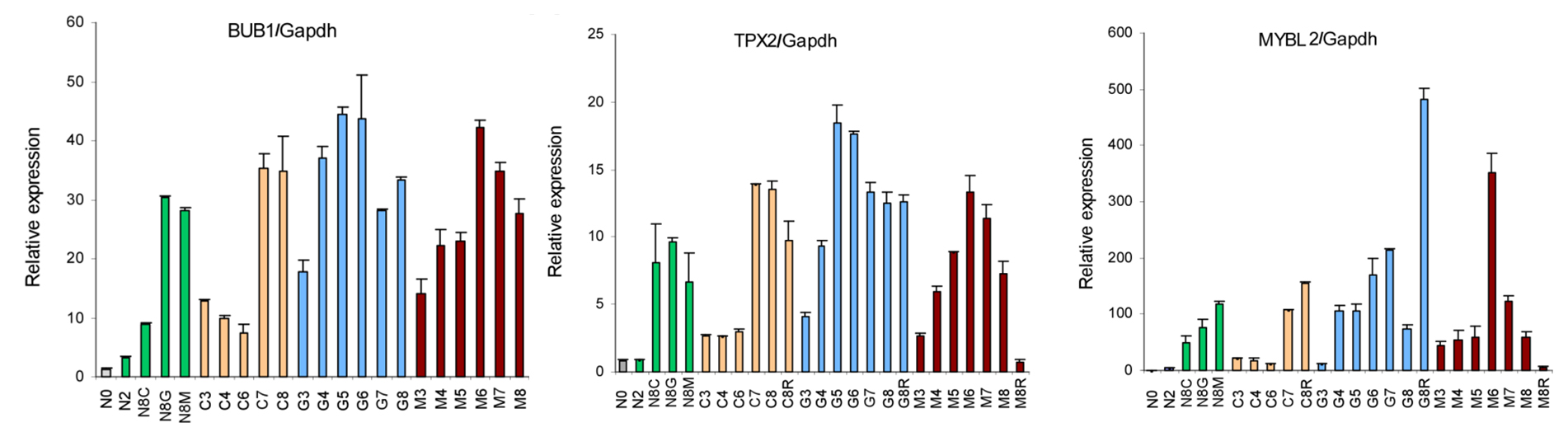

Supplement: Figure S3 — QRT-PCR validation in proliferation cluster genes: BUB1 TPX2 and MYBL2 at progressive time points along the long term in vitro culture. (3.01 MB TIF) [file pone.0014632.s003.tif]

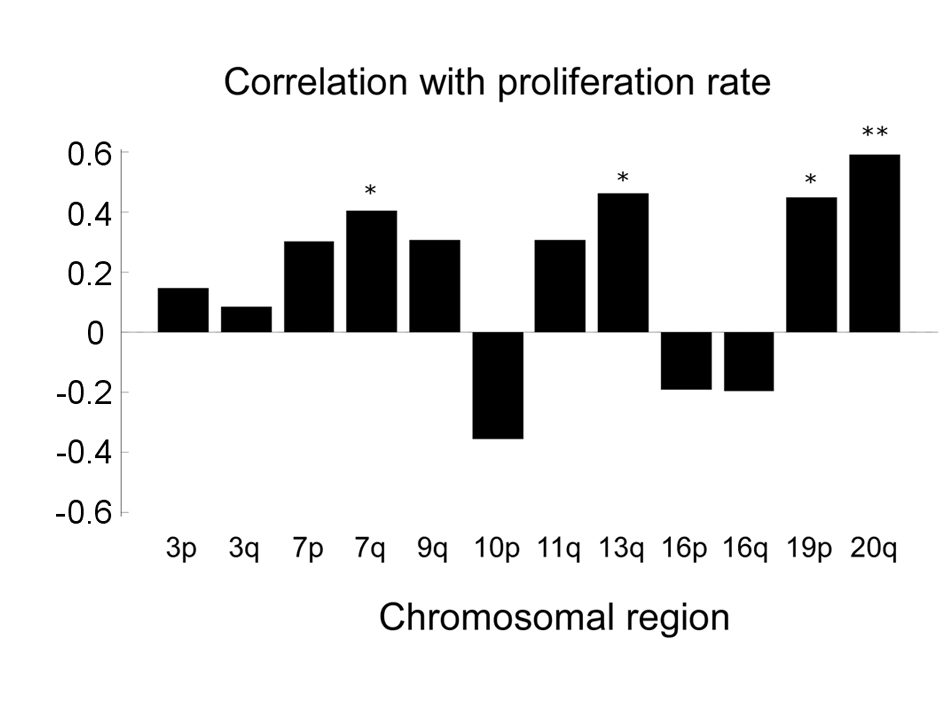

Supplement: Figure S4 — Pearson correlation between the growth rate and the expression of genes on each indicated chromosomal arm. * p-value<0.05, ** p-value<0.001 for up regulation of genes encoded by this region. (0.69 MB TIF) [file pone.0014632.s004.tif]

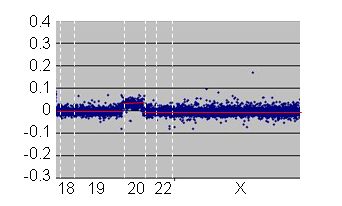

Supplement: Figure S5 — Raw array CGH data for WI-38 human diploid fibroblast cells obtained from passage 93 in an in-vitro transformation experiment (Milyavsky et al. 2005). Around 25% percent of the cells contained amplification of chromosome 20. (0.24 MB TIF) [file pone.0014632.s005.tif]

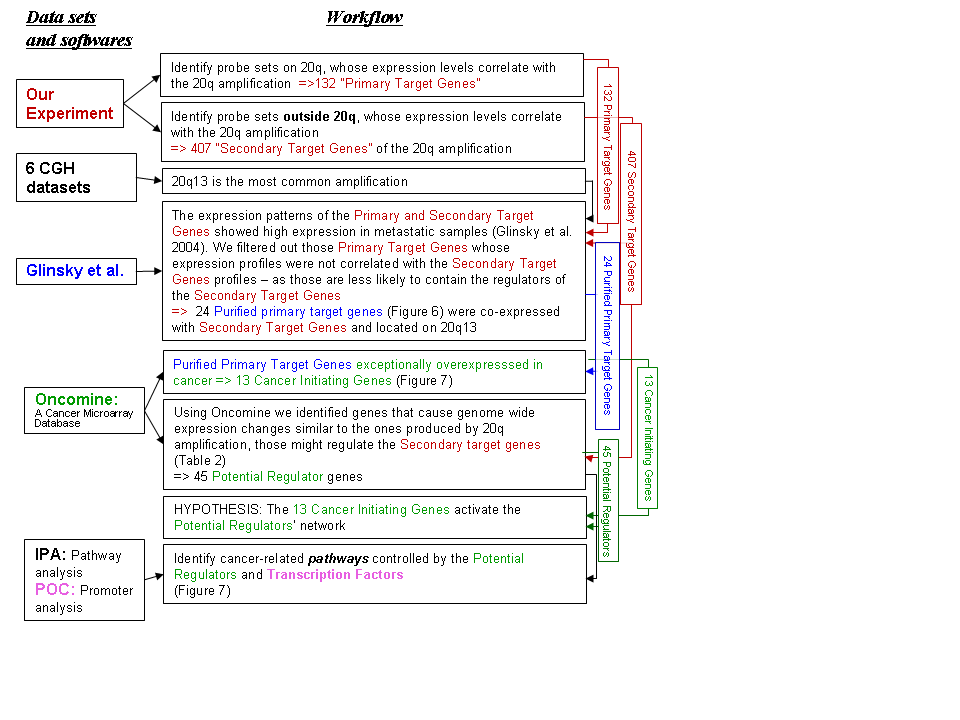

Supplement: Figure S6 — Workflow of our analysis of the tumorigenic effect of 20q amplification. The colors represent four different data types that were used: Oncomine (green), our prostate in-vitro transformation model (red), prostate samples from patients (blue) and array CGH data (black). Each box represents a step in the analysis. The arrows indicate that information was passed from one box to another. (0.09 MB TIF) [file pone.0014632.s006.tif]

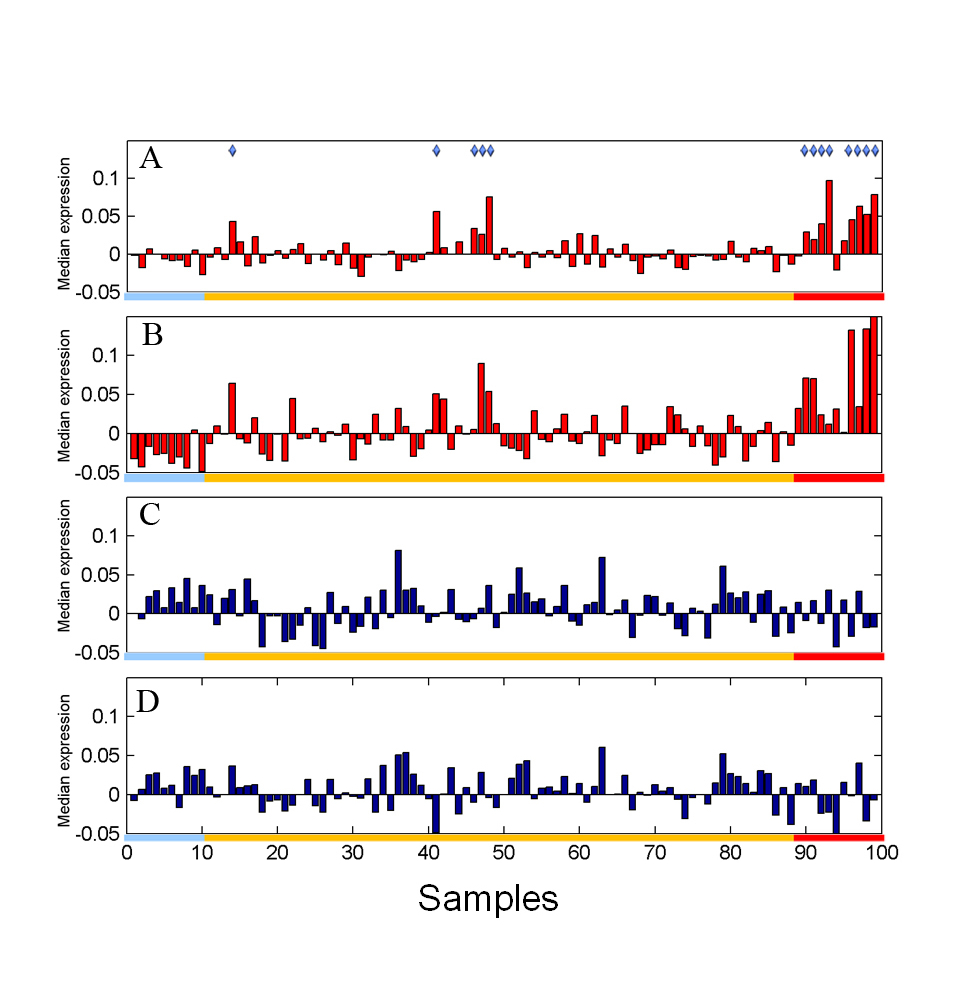

Supplement: Figure S7 — Expression pattern of 20q genes in normal and cancerous prostate samples. Median Expression pattern (after normalization and log2) of genes in 99 in-vivo normal and cancerous prostate samples (Glinsky et al. 2004) at different stages of progression, as indicated by the color bar (blue for normal prostate, orange for primary tumor, red for metastatic tissue). A. Median expression of our "primary target genes". The blue dots mark samples which we predict to have 20q amplification B. Median expression of the "secondary target genes". C. Median expression of the genes encoded by the 20p chromosomal region. D. Median expression of the 20q genes which were not identified as "primary target genes" (not correlated with our 20q expression karyotype). (2.91 MB TIF) [file pone.0014632.s007.tif]

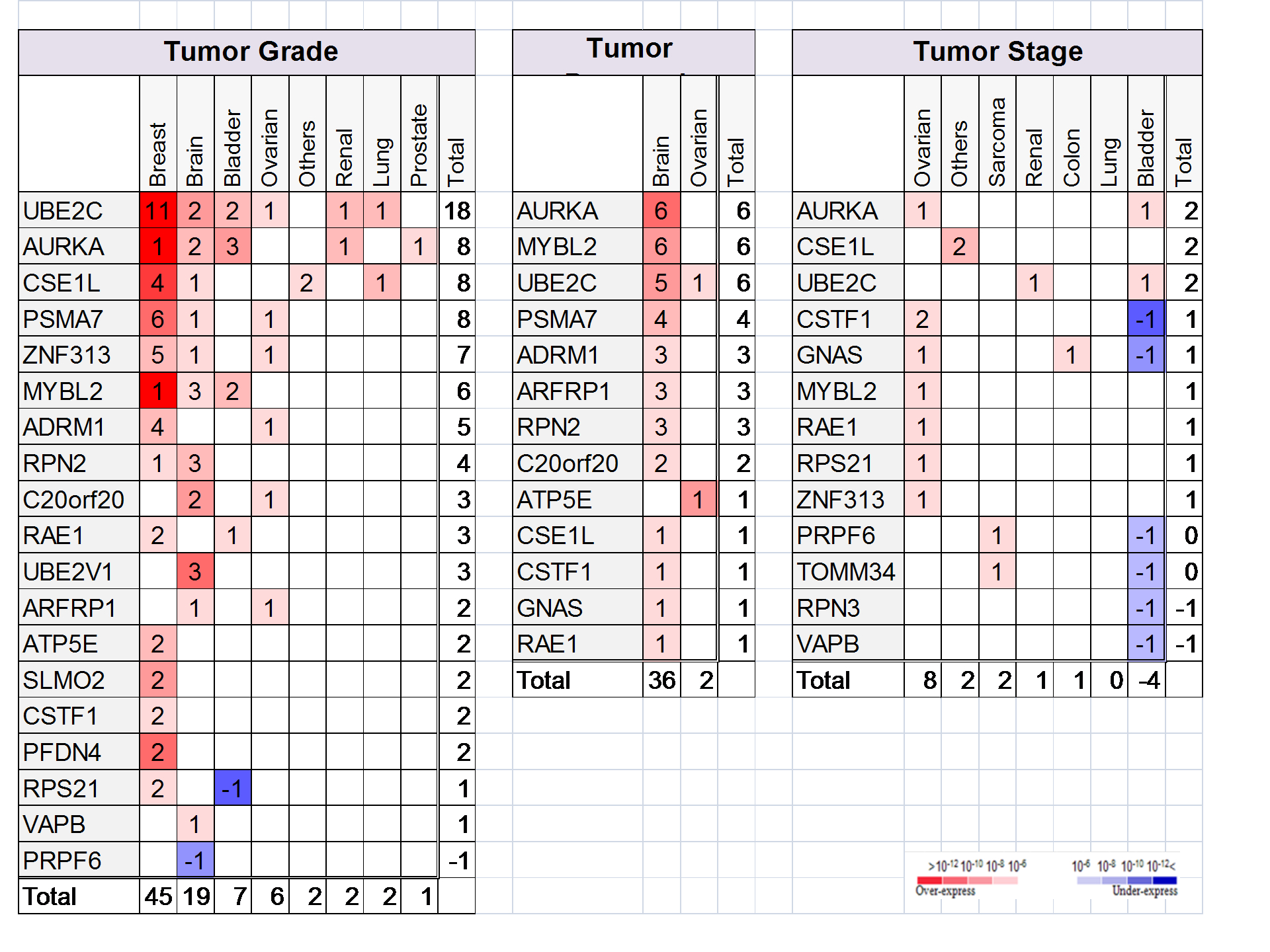

Supplement: Figure S8 — Summary of the expression of our 24 "purified primary target genes" in more than 360 experiments of different cancers. The colors represent the p-value (as calculated by Oncomine) for significant over-expression (red colors) or under-expression (blue colors) in different grade, stage or prognosis of each single "purified primary target genes". If there are several experiments (denoted by the number in the corresponding box) the color represents the best p-value from these experiments. (8.32 MB TIF) [file pone.0014632.s008.tif]

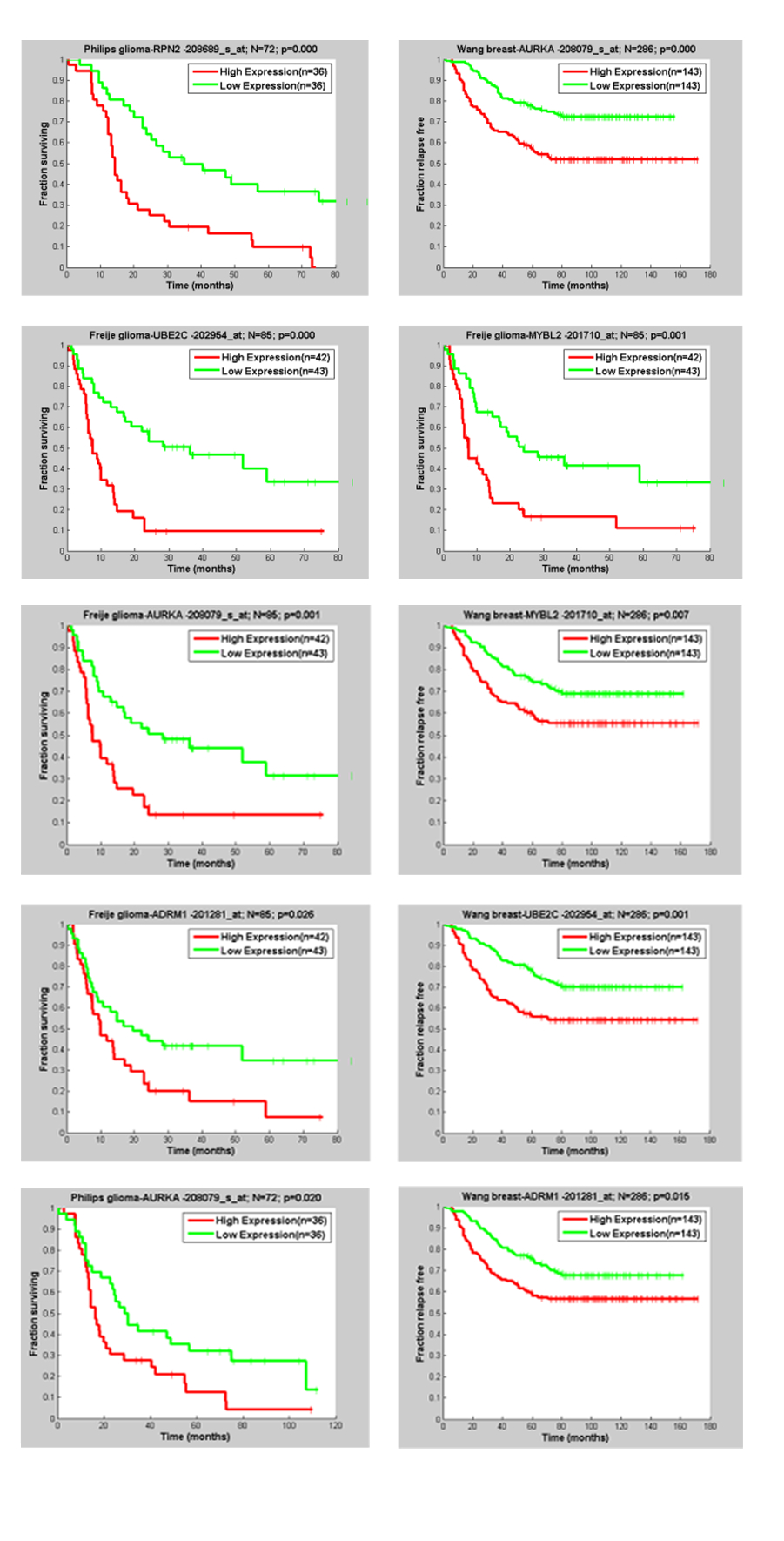

Supplement: Figure S9 — Survival and relapse test for our "cancer initiating genes" in several data sets. The figure shows genes whose expression levels (high 50% vs. low) significantly differentiate cancer patients by survival and relapse in several studies (4.81 MB TIF) [file pone.0014632.s009.tif]
