# Supplementary material for: Amplification of the 20q Chromosomal Arm Occurs Early in Tumorigenic Transformation and May Initiate Cancer
Source: PLoS One. 2011 Jan 31;6(1):e14632. doi: 10.1371/journal.pone.0014632 (PMC3031497; doi:10.1371/journal.pone.0014632)
Supplement: Text S1 — Supplementary information (0.16 MB DOC) [file pone.0014632.s012.doc]

**Amplification of the 20q chromosomal arm occurs early in tumorigenic transformation and may initiate cancer.**

**Text S1**

### The Proliferation cluster

### We previously identified, in in-vitro transformed normal human fibroblasts, a gene expression profile represented by 168 cell cycle–regulated genes that have been termed "the proliferation cluster" (Tabach et al. 2005). Increased expression of many of these genes, is often associated with poor outcome (Rosty et al. 2005; Salvatore et al. 2007; Whitfield et al. 2006) and with aneuploidy (Carter et al. 2006). In the current work, we identified a cluster of 177 transcripts (Figure 2D, top) which had high overlap with the "proliferation cluster" (52 genes out of the 177). We examined the expression of these genes in 99 samples, obtained from normal prostate, tumor and metastasis of prostate cancer patients (Glinsky et al. 2004). The genes showed significant up regulation from normal to tumor (p-value = 0.00024 using t-test) to metastasis (p-value = 0.00013) (Figure 2D, bottom). As expected, this group includes mainly genes associated with different aspects of cell proliferation such as DNA replication (RFC2, CDC7, MCMs, PCNA, POLA etc.), regulation of cell cycle progression (CDC2, CCNB1, CCNB2, MYBL2, CDC25a, CCNE2, CCNA2), DNA repair (BRCA1, RAD51, MSH2) and the regulation of mitotic spindle assembly (CDC20, PRC1, TPX2, BUB1, BUB1B, AURKA etc.). To validate the trend of expression observed by microarrays, we performed real time QRT-PCR on three representative genes, TPX2, MYBL2 and BUB1 (see Figure S3).

There was marked up regulation of proliferation genes with time in culture. This observation is in agreement with an acceleration of cell growth rate (Figure 2A), down regulation of p16INK4a (Figure 2B) and higher proportion of proliferating cells in later passages (as judged by BrdU labeling) (Figure 2B bottom panel). These observations suggest that during prolonged cultivation of EP156T prostate immortalized cells a selection process took place favoring survival and expansion of cells with higher proliferative capacities, which might lead to a transformed phenotype.

### Transcriptional decline of cell adhesion and glycoprotein genes along the transformation process

### Our clustering analysis identified a cluster of 296 genes whose expression was negatively correlated with the proliferation cluster (Figure 2D, top). Using the data from (Glinsky et al. 2004), we found that these genes were significantly down regulated in metastasis samples (p-value =0.00023) compared to primary tumors, and in the latter, when compared to normal samples (p-value =0.00013) (Figure 2E, bottom).

When examining the Gene Ontology (GO) enrichment using the David software (Dennis et al. 2003), several GO categories were found to be significantly over-represented in this cluster: [glycoprotein](http://us.expasy.org/cgi-bin/get-entries?KW=glycoprotein)s (p-value 2.4*10-20), [extracellular region](http://www.ebi.ac.uk/ego/DisplayGoTerm?id=GO:0005576) genes (p-value 4.3*10-13) and [cell adhesion](http://us.expasy.org/cgi-bin/get-entries?KW=cell adhesion) (p-value 2*10-10). This cluster also includes significantly genes (collagen, laminin, vav3 oncogene, FN1, MYL9, MYLK, CD44, etc) associated with different KEGG pathways of cell-cell interactions, such as [Cell Communication](http://david.abcc.ncifcrf.gov/kegg.jsp?PATH=hsa01430$Cell Communication), [ECM-receptor interaction](http://david.abcc.ncifcrf.gov/kegg.jsp?PATH=hsa04512$ECM-receptor interaction) and [Focal Adhesion](http://david.abcc.ncifcrf.gov/kegg.jsp?PATH=hsa04510$Focal adhesion) (p-value< 0.0006). Down regulation of the same gene set in an in vitro model of prostate carcinogenesis and in advanced prostate cancer samples suggests that our model is able to recapitulate molecular events which occur during in vivo malignant transformation of the prostate gland.

***The Chromosomal Imbalances Analysis (CIA)***

Our working hypothesis is that changes in the copy number of the DNA of a full chromosome or part of it are reflected in the gene expression. Deletion of a chromosome causes, on the average, down regulation of the genes of that chromosome, while duplication should be reflected by a higher expression level. In order to identify chromosomal imbalances, we compared expression levels of the genes that reside on each chromosomal arm, in each sample, to a reference sample of normal cells (N0), and searched for significant differences in the expression levels, using paired t-test, as explained below.

Our input is the log expression levels of n genes g on chromosomal arm C of the reference sample N0 and of one of the experimental samples S. The paired t-test determines whether the expression levels of these n genes differ between the two samples in a significant way. We are working under the null hypothesis that the paired differences, Δg, are independent and identically normally distributed. We calculate the statistic


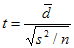


where

$$\bar{d}=\sum_{g}^{n} \Delta_{g}/n$$

is the mean difference of expression of the genes on chromosome C between the samples N0 and S, s² is the variance of the Δg, and t is a Student t quantile with n-1 degrees of freedom.

For every sample S and chromosome C we calculate the p-value Pc(S) for falsely rejecting the null hypothesis. When the p-value Pc(S) is below a certain threshold, we identify an amplification or deletion, and calculate a quantity Fc(S) that represents the magnitude of the copy-number change. We chose the median of the expression differences Δg between the N0 and S on chromosomal arm C as this quantity:

Fc(S) = median(Δg)

When there were two or more probe sets for the same gene (defined as those with the same gene symbol), their expression levels were combined as described in detail in Hertzberg at al (Hertzberg et al. 2007). Briefly, when the probesets were correlated (Pearson correlation > 0.5), we used the average expression of the gene's probesets as the expression level of the gene. In the case when the probesets where not correlated, we used only one representative probeset. We preferred the probeset that ended with '_at'; if no such probesets existed; we used probesets that ended with ‘_s_at’ or ‘_x_at’. After this process, each gene symbol had one expression value in each sample. From the total number of ~12,000 unique genes, we decided arbitrarily to take the threshold that maximized the number of p-values that were less than 0.005. These 7000 most variable genes were chosen for the analysis (i.e. the number of genes of each chromosomal arm was set by including the genes of the arm that belonged to these 7000).

### Comparison of the two computational methods

Two methods have been used to identify "expression karyotype" in samples. Using the paired t-test approach we calculated, for each tested sample S, a P-value for each chromosome to test if the mean difference between the genes in N0, compared to sample Si, is different from zero. The binomial approach (Hertzberg et al. 2007) determines if a significant fraction of the genes on the chromosome is over-expressed in S versus N0 (or under expressed). The two approaches are different in two main aspects: 1) Hertzberg et al use binomial distribution, while the other approach uses the paired t-test and has much more statistical power when the difference between groups is small, relative to the variation within groups. 2) While Hertzberg et al worked with all the genes on the chromosome we decided to examine the influence of using the most variable genes on the p-value obtained from the paired t-test. One can see from figure S1 that using all the genes reduces the signal that was obtained from the paired t-test. We can clean the signal and remove irrelevant genes by choosing the 5500-7500 most variable genes. The exact number of genes used has small influence on the results of the analysis as long as we are in the range of 5500-7500 genes. The result of the paired t-test method can be seen in Supplementary Figure S1.

The SKY results were used to estimate the predictive power of the two computational methods. The SKY results were summarized as follows that if we observed in more than 50% of the cells the same chromosomal aberration, we considered it as a true aberration; otherwise we attributed the observed aberration to noise and removed it from the analysis. Based on these results we compared the two computational methods using receiver operating characteristics (ROC) analysis (see Figure S2). ROC analysis is one of the most widely used methods for summarizing intrinsic properties of a diagnostic system, and is often used in evaluation and comparison of diagnostic methods. The ROC curve was done as described in Hertzberg et al. (2007) and as we can see, the performances of the two methods were similar.

***A detailed description of our model for cancerous transformation induced by 20q amplification.*** We first present a brief overview of the steps which were taken in order to better understand the impact of 20q amplification on our in vitro model, as depicted in Figure S6; in what follows, a subsection will be devoted to each box in the figure. The workflow is presented in the same order as the boxes appear in the chart in Figure S6. The colors in the figure are related to the databases that were used, as shown on the left side.

To resolve the cancer promoting effect of 20q amplification, our first aim (Figure S6, first box) was to identify those probesets on 20q, whose expression levels were correlated with the 20q amplification (as derived from our Chromosomal Imbalances Analysis algorithm, see Methods). Thus, we generated a list of "primary target genes" containing 132 probesets from 20q that the amplification of 20q probably increased their expression. We believe that the "primary target genes" might contain genes that initiate and promote cancer. In what follows, we apply various filters and operations to identify a subset of likely "cancer initiating genes" among these 132. Similarly, 407 probesets of "secondary target genes" were identified (Figure S6, second box); these are probesets located on chromosomal arms other than 20q, but whose expression levels correlated with 20q amplification, and as such their expression is also likely to be influenced by the amplification of 20q probably by the "cancer initiating genes". We assume that the "secondary target genes" are the main vehicle through which the "cancer initiating genes" induce the transformation. The aim of the next steps is to narrow down the list of "cancer initiating genes". Two main filters were used. First, we identified 20q13, from six independent array CGH data sets, as the region on 20q, which is most commonly amplified in cancer (Figure S6, 3^rd^ box). Second, we selected only those "primary target genes" from 20q13 whose expression, measured in (Glinsky et al. 2004) was correlated (Figure S6, 4^th^ box) with *med("secondary target genes")* and thus, their increased expression was likely to have affected the "secondary target genes". These two filtering operations reduced the number of "primary target genes" probe sets from 132 to 34, corresponding to 24 unique genes. The next step (Figure S6, 5^th^ box) was to identify among these 24 genes a subset of genes that were over-expressed in cancer; the Oncomine database was used for this. The resulting 13 genes were termed our "cancer initiating genes". We describe now in detail the procedures used to generate the data described in each box.

***Primary targets of the amplification (Figure S6, 1^st^ box).*** First we tested which of the genes located on 20q had altered expression following the chromosomal aberration. Using our expression karyotype we identified 132 probesets encoded on 20q with high correlation (R>0.6) between their expression fold change and the median fold change of 20q (see figure 3 for 20q median fold change and Methods). These 132 probe sets comprise one third of the genes on 20q: we refer to them as the *"primary target genes"* of the amplification.

***Secondary target genes (Figure S6, 2^nd^ box).*** Next, we turned to genes outside 20q and found 407 probesets with high correlation (R>0.7) between their fold change (compared to N0) and the median fold change of 20q (see Figure 3 for 20q median fold change, and Methods). We termed these as "secondary target genes" of the 20q amplification. Since the threshold value of R>0.7 was chosen arbitrarily, we changed the threshold; 1353 probesets passed with R>0.6. The entire analysis was repeated using this definition of "secondary target genes", and since the two definitions yielded similar results (see below), we stayed with the shorter list, of 407 "secondary target genes".

***Gene expression in prostate cancer (4^th^ box).*** To ascertain the importance of "primary target genes" and "secondary target genes" in cancer, we examined the median expression patterns of both groups in an independent dataset of 99 in vivo normal and cancerous prostate samples (Glinsky et al. 2004) at different stages of progression. Both gene groups showed high expression in metastatic samples and low expression in normal samples (Supplementary Figure S7 A-B). The median expression levels of "primary target genes" and "secondary target genes" were significantly correlated with each other (Pearson R = 0.66). Importantly, genes encoded by the 20p chromosomal region, as well as those 20q genes which were not "primary target genes" showed poor correlation to "secondary target genes" or to stage (normal, cancer and metastasis) in these data (Supplementary Figure S7 C-D). Our finding that the expression of "primary target genes" and "secondary target genes" was up regulated in an in vitro transformation model and also in metastatic samples suggests that these genes are biologically related and may play important roles in the process of cancer progression.

***"Purified primary target genes" (Figure S6, 3^rd^ and 4^th^ boxes)****.* Not all the 132 "primary target genes" were co-expressed or correlated with the "secondary target genes" in the Glinsky et al datasets (Glinsky et al. 2004). In order to filter out "primary target genes" whose role in cancer is in doubt, we applied two filters. First, we restricted our analysis only to those "primary target genes" whose expression profiles were correlated with the median "secondary target genes" profile in the Glinsky et al. datasets (R> 0.3, and FDR < 0.1) since we believe these correlated "primary target genes" are more likely to contain the regulators of the "secondary target genes". Second, to eliminate reliance on one particular data set, we examined previously published array CGH data from several cancer types (Hodgson et al. 2003; Huret et al. 2003; Kimura et al. 2004; Korn et al. 1999; Mahlamaki et al. 2002; Tanner et al. 2000), and identified 20q13 as the most common region of copy number alterations. Therefore we restricted our list of "purified primary target genes" to 34 probesets (belonging to 24 genes) that were located on 20q13, were co-expressed and correlated with "secondary target genes" in the Glinsky prostate cancer dataset and had increased expression as a result of amplification of 20q in our data.

***Analysis of individual "purified primary target genes" using Oncomine (Figure S6, 5^th^ box).*** We analyzed the expression profile of each of the 24 "purified primary target genes" using Oncomine (Rhodes et al. 2004), a compendium of expression data from 25,812 microarrays measured for 361 cancer data sets. Out of the 24 "purified primary target genes", 23 were over-expressed in cancer tissues (versus normal, see Figure 6). 18 were also over-expressed in higher malignant grades, when compared to low grades of cancer; 13 were over-expressed in more progressed (versus early) malignant stages, and 13 were over-expressed in bad (compared to good) prognosis samples (see Figure S8 for stage, grade and prognosis). Thirteen out of the 24 "purified primary target genes": UBE2C, ADRM1, CSE1L, RPN2, C20orf45, MYBL2, TOMM34, AURKA, RAE1, PFDN4, PSMA7, RPS21 and VAPB showed exceptionally significant over-expression in several cancers. These genes were filtered from the 132 probe sets on 20q, they correlate with 20q amplification, over-expressed in cancer, related to stage, grade and prognosis, and found to be related, by array CGH, to frequent 20q13 amplifications. Genes like AURKA, UBE2C, TOMM34, CSE1L and MYBL2 were suggested before as potential oncogenes, while ADRM1, RPN2 and C2orf45 were barely discussed in the literature. We speculate that the over-expression of these genes might have an important oncogenic effect. Interestingly, a very recent paper (Carvalho et al. 2009) identified 7 genes on 20q, whose amplification may play an oncogenic role in colon cancer: C20orf24, C20orf20, AURKA, TH1L, ADRM1, RNPC1 and TCFL5. C20orf24 is not on 20q13, 4 of the remaining 6 are among our 24 "purified primary target genes" and two are in our list of "cancer initiating genes".

***Determining "potential regulator genes" using the Oncomine database (Figure S6, 6^th^ box).*** The Oncomine database (Rhodes et al. 2004) contains a large number of gene sets, called "concepts". Concepts are defined as a set of genes (a gene “signature”) that is characteristically associated with some biological phenomenon (e.g., differential expression in disease or after drug treatment, or genes involved in a specific pathway or regulatory mechanism). For example, the concept "Human Mammary Epithelial Cells Oncogene Transfection - Top 10% over-expressed in *c-Myc*" was obtained by comparing cells with normal *c-Myc* expression level and similar cells in which *c-Myc* was induced or over-expressed, and ranking the differentially expressed genes by their fold change. The database contains 12000 such concepts; for each concept we performed a hypergeometric test for overlap with the "secondary target genes"; 584 concepts (see Table S1 for the full list) passed at False Discovery Rate (FDR) of less than 0.01. We then searched manually the definitions of these 584 concepts for those that were associated with any known cancer related gene; 110 such concepts were found. For example the *c-Myc* related concept mentioned above had the most significant overlap (P-value = 3.8*10-59 using hyper geometric test) with our "secondary target genes"; according to our interpretation, this suggested that *c-Myc* or key factors in its regulatory network became activated as a result of the 20q amplification, and caused up-regulation of a significant part of our "secondary target genes". In case of anticancer genes we expected the opposite phenomenon. It is important to note that from the 584 significant Oncomine concepts we identified and selected only those 110 that were obtained from comparing samples with normal expression of a wild type cancer related gene, with samples in which the same gene was activated, over-expressed or in some cases deleted. Each of these 110 concepts is associated with a "potential regulator gene", and since many "potential regulator genes" appear in more than one concept, we finally have a group of 45 distinct "potential regulator genes" genes that are summarized in Table 2.

We repeated this analysis using a correlation threshold of R>0.6 (instead of R>0.7) to define the "secondary target genes" (as mentioned above). 1353 probesets passed this lower threshold; using them as our "secondary target genes" in the Oncomine-based analysis we got 1002 significant concepts (at FDR<0.01), instead of 584; 521 of these were identical to the concepts identified using "secondary target genes" based on R>0.7. Only 7 new "potential regulator genes" were added; out of these added "potential regulator genes" only IL6 was found in more than one concept and with an impressive p-value <4.7*10^-12^. The similarity of the final list of "potential regulator genes" shows that our analysis is robust against varying the threshold used for selection of the "secondary target genes".

The most significant overlaps with our "secondary target genes" were the concepts associated with activity of *c- MYC*, Mutant *p53*, *MLL* and the loss of activity of Estrogen Receptor, *TEL-AML* translocation, B-Catenin and *ERBB2*.

***Relating the cells cultured in vitro to prostate cancer.***

Our current study examined "real time" changes in normal cells as their gene expression, karyotype and proliferation rate gradually changed and they acquired a premalignant phenotype along the process. After 650 days in culture our premalignant hTERT-immortalized prostate epithelial cells shared several phenotypic, chromosomal and transcriptional attributes with prostate cancer samples. Primary epithelial cells from human prostate are known to be slow growing and could create xenografts only when immortalized using viral sequences (Van Bokhoven 2004) that are known to inactivate the p53 and the pRb tumor suppressor pathways. As a result, and as expected from other studies (Choo et al. 1999; Kaighn et al. 1989; Webber et al. 1996; Weijerman et al. 1994; Yasunaga et al. 2001) our premalignant cells failed to generate xenografts in mice.

The ultimate test for completion of the malignant transformation is generating cancerous xenografts in mice. Our attempts at subcutaneous implantation of cells from the different (N,C,G and M) lines unsurprisingly did not generate growing tumors in mice, as discussed above. For this reason we refer to the cells in our cultures as partially transformed or premalignant. This nomenclature is justified since we have shown above that several properties measured for our in vitro cell cultures were also observed in human prostate cancer samples (Glinsky et al. 2004). In particular, the similar behavior of the genes from the proliferation cluster and the cell adhesion/glycoprotein associated cluster should be noted, together with the increasing proliferation rate and colony formation, all characteristic of prostate cancer evolution from premalignant to primary tumor and to metastatic phenotypes.
